# *SST* gene hypermethylation acts as a pan‐cancer marker for pancreatic ductal adenocarcinoma and multiple other tumors: toward its use for blood‐based diagnosis

**DOI:** 10.1002/1878-0261.12684

**Published:** 2020-04-14

**Authors:** Mehdi Manoochehri, Yenan Wu, Nathalia A. Giese, Oliver Strobel, Stefanie Kutschmann, Florian Haller, Jörg D. Hoheisel, Evgeny A. Moskalev, Thilo Hackert, Andrea S. Bauer

**Affiliations:** ^1^ Division of Functional Genome Analysis German Cancer Research Center (DKFZ) Heidelberg Germany; ^2^ Molecular Genetics of Breast Cancer German Cancer Research Center (DKFZ) Heidelberg Germany; ^3^ Department of General Surgery University Hospital Heidelberg Germany; ^4^ Diagnostic Molecular Pathology Institute of Pathology Friedrich‐Alexander University Erlangen Germany

**Keywords:** biomarker, DNA methylation, gene expression, liquid biopsy, pancreatic cancer, solid tumors, somatostatin

## Abstract

Aberrant DNA methylation is often involved in carcinogenesis. Our initial goal was to identify DNA methylation biomarkers associated with pancreatic cancer. A genomewide methylation study was performed on DNA from pancreatic ductal adenocarcinoma (PDAC) and endocrine pancreas tumors. Validation of DNA methylation patterns and concomitant alterations in expression of gene candidates was performed on patient samples and pancreatic cancer cell lines. Furthermore, validation was done on independent data from The Cancer Genome Atlas (TCGA) and Gene Expression Omnibus (GEO). Finally, droplet digital PCR was employed to detect DNA methylation marks in cell‐free (cf) DNA isolated from plasma samples of PDAC patients and cancer‐free blood donors. Hypermethylation of the *SST* gene (encoding somatostatin) and concomitant downregulation of its expression were discovered in PDAC and endocrine tumor tissues while not being present in chronic pancreatitis (inflamed) tissues and normal pancreas. Fittingly, treatment with a somatostatin agonist (octreotide) reduced cell proliferation and migration of pancreatic cancer cells. Diagnostic performance of *SST* methylation in a receiver operating characteristic curve analysis was 100% and 89% for tissue and plasma samples, respectively. A large body of TCGA and GEO data confirmed *SST* hypermethylation and downregulation in PDAC and showed a similar effect in a broad spectrum of other tumor entities. *SST* promoter methylation represents a sensitive and promising molecular, pan‐cancer biomarker detectable in tumor tissue, and liquid biopsy samples.

## Graphical abstract

Hypermethylation of the *SST* gene (encoding somatostatin) and concomitant downregulation of its expression were found to be common in tumor entities, representing a promising pan‐cancer biomarker. Somatostatin agonist octreotide reduced proliferation and migration of pancreatic cancer cells, indicating a tumor‐suppressive function of somatostatin. Variation in *SST* methylation was detectable in not only tumor tissues but also patients’ plasma samples.

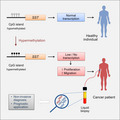

**Abbreviations**
cfDNA: cell‐free DNA
COAD: colon adenocarcinoma
COBRA: combined bisulfite restriction analysis
CP: chronic pancreatitis
CT: cystic tumors
ddPCR: droplet digital PCR
DMR: differentially methylated region
ESCA: esophageal carcinoma
FDR: false discovery rate
GEO: Gene Expression Omnibus
GTEx: Genotype‐Tissue Expression
HNSC: head neck squamous cell carcinoma
IPA: ingenuity pathway analysis
N.PDAC: peritumoral tissue next to PDAC
NET: pancreatic neuroendocrine tumor
PDAC: pancreatic ductal adenocarcinoma
READ: rectum adenocarcinoma
ROC: receiver operating characteristic
STAD: stomach adenocarcinoma
TCGA: The Cancer Genome Atlas

## 1 Introduction

Pancreatic ductal adenocarcinoma (PDAC) is the currently fourth leading cause of cancer‐related death in developed countries, with a 5‐year survival rate of about 6% (Rahib *et al.*, 2014; Siegel *et al.*, 2019). It is predicted to rise and take second place only to lung cancer by year 2030. PDAC is mostly diagnosed very late; one reason for this is the lack of appropriately informative diagnostic tools. In addition, metastasis occurs very early and aggressively in PDAC, leading to a rather dismal overall prognosis (Costello *et al.*, 2012). Although there have been reports on potential biomarkers that might be helpful in diagnosis, prognosis, and prediction of PDAC, basically none has made it into clinical practice in recent years (Dutta *et al.*, 2012; Fong and Winter, 2012; Nolen *et al.*, 2014; Winter *et al.*, 2013). Also epigenetic modifications, in particular DNA methylation, have been reported as biomarkers to detect pancreatic cancer in early stages of the disease (Bydoun *et al.*, 2018; Kisiel *et al.*, 2015; Nones *et al.*, 2014; Yi *et al.*, 2013). Aberrant alterations of DNA methylation patterns usually occur in CpG islands (CGI) and could play a role in the inactivation of tumor suppressor genes, activation of oncogenes, and altering the integrity of the genome (Irizarry *et al.*, 2009). Several genes have been reported to be differentially methylated in PDAC tissue compared to that of normal pancreas (Lee *et al.*, 2019; Nones *et al.*, 2014; Vincent *et al.*, 2011), suggesting utility for diagnostic purposes.

In this study, we performed a genomewide DNA methylation study to identify DNA methylation marks that could be applicable for PDAC diagnosis. Candidates were identified by analyzing clinical tissue samples of PDAC, pancreatic neuroendocrine tumor (NET) for comparison, chronic pancreatitis (CP), noncancerous pancreas tissue, and noncancerous peritumoral tissue surrounding PDAC (N.PDAC). NETs originate from neuroendocrine cells, while PDAC arises from exocrine cells, and are relatively rare with about 1–2% of all pancreatic neoplasms. Their incidence has increased during recent decades (Zhou *et al.*, 2012), however. The peritumoral tissue surrounding PDAC has been described as being more similar at the transcript level to PDAC than to healthy noncancerous tissue (Bauer *et al.*, 2018). This may actually be a reason for the miserable prognosis of PDAC as compared to cystic tumors (CT), whose peritumoral tissues are basically very similar to healthy tissue. We then validated and verified the methylation status using additional clinical samples. Furthermore, additional studies on other tumor tissues were performed using data available at the The Cancer Genome Atlas (TCGA) and Gene Expression Omnibus (GEO) databases. Finally, we investigated hypermethylation marks in cell‐free DNA (cfDNA) in plasma samples from pancreatic cancer patients and relevant controls (for a graphical overview of the work, see Fig. S1).

## 2 Materials and methods

### 2.1 Tissue samples and patient cohort

Human pancreatic tissue samples were collected during surgery. In all cases, written informed consent had been obtained from the patients. The study was approved by the Ethics Committee Heidelberg, Germany, and performed in compliance with the provisions of the Declaration of Helsinki. Demographic and clinical characteristics of the patient study population are shown in Table S1. The frozen tissues were sectioned with a cryotome; three sections from each tumor sample were used for histopathology. Various clinical parameters of the patients including the age, survival time, metastasis, and tumor stage were taken into account (Bauer *et al.*, 2018).

### 2.2 DNA/RNA isolation

Genomic DNA and total RNA were isolated from fresh‐frozen tissue samples with the AllPrep Isolation Kit (Qiagen, Hilden, Germany), following the protocol suggested by the manufacturer. RNA integrity was evaluated using an Agilent 2100 Bioanalyzer (Agilent Technologies, Palo Alto, CA, USA). DNA quantities and qualities were determined using a NanoDrop 1000 spectrophotometer (ThermoFisher Scientific, Waltham, MA, USA).

### 2.3 In‐house gene expression data

Expression profiling data are available from extensive previous studies (Bauer *et al.*, 2018). In summary, there are data on 195 cases of PDAC, 30 cases of peritumoral tissue from next to PDAC (N.PDAC), 24 CT, 22 peritumoral tissues from next to cystic tumors, 59 samples of CP, and 41 healthy pancreatic tissues from noncancer patients (N). Total RNA from individual samples was analyzed on the Sentrix Human‐6v3 Whole Genome Expression BeadChips (Sentrix Human WG‐6; Illumina, San Diego, CA, USA). Differential expression analysis was performed using the *LIMMA* package by pairwise comparisons of the groups (Ritchie *et al.*, 2015). The resulting *P*‐values were adjusted for multiple testing using Benjamini–Hochberg's false discovery rate (FDR) method; features with a FDR < 0.01 and an absolute log_2_‐fold change (log_2_FC) > 0.5 were considered significant.

### 2.4 DNA methylation profiling

Genomewide DNA methylation analysis was performed using the Illumina Infinium 450k DNA methylation platform (Illumina) on 26 PDAC tissues, 24 normal pancreases, 12 CPs, 12 NETs, and 12 peritumoral samples close to PDAC. The analysis procedure followed the manufacturer’s standard workflow. The resulting raw data were preprocessed using the RnBeads routine (Assenov *et al.*, 2014) at default settings. Quality control, probe filtering, background correction, batch effect correction, and selection of differentially methylated regions (DMRs) were performed as recommended. DMRs were selected from the list of Infinium probes that passed quality control assessments (FDR‐adjusted *P* ≤ 0.01, absolute methylation difference ≥ 0.15). The data are available at Table S2.

### 2.5 Pathway analysis and candidate selection

The top genes corresponding to the significantly hypermethylated DMR candidates were analyzed with ingenuity pathway analysis (IPA) software (Qiagen, Redwood City, CA, USA) and ConsensusPathDB (Herwig *et al.*, 2016). For the selection of final candidates, we focused on anticorrelated (hypermethylated at DNA level and downregulated at RNA level) genes in PDAC vs. normal pancreas samples. Of these anticorrelated genes, protein encoding genes were selected for further analyses that are involved in cancer‐related pathways.

### 2.6 Gene‐specific methylation analysis

For verification and validation of the DMRs obtained from the discovery phase, combined bisulfite restriction analysis (COBRA) was performed. (Xiong and Laird, 1997). In short, 1 µg of DNA was treated with sodium bisulfite (EpiTect Bisulfite Kit; Qiagen, Germany) following the manufacturer’s protocol. Amplification was performed using specific primer pairs (Table S3) and HotStarTaq DNA Polymerase (Qiagen) as described (Moskalev *et al.*, 2011). Bisulfite sequencing was performed to confirm the methylation in the CGI region of the *SST* gene promoter. The sequencing data were analyzed using the biqanalyzer software (Agilent Technologies, Palo Alto, CA, USA) (Bock *et al.*, 2005) .

### 2.7 Bisulfite pyrosequencing

Quantitative validation of DNA methylation was performed using bisulfite pyrosequencing according to a protocol described previously (Moskalev *et al.*, 2015). The sequences of all primers are listed in Table S3. The software pyromark q24 v.2.0.6 (Qiagen) was used for quantification of CpG methylation percentages. The amplification bias toward unmethylated alleles was corrected using the calibration data derived from a set of EpiTect control DNA samples of 0%, 25%, 50%, 75%, 100% methylation (Qiagen) and cubic polynomial regression as described (Moskalev *et al.*, 2011).

### 2.8 Real‐time qPCR

The relative quantification of *SST* gene expression was performed by real‐time qPCR on a Light Cycler 480 instrument (Roche Diagnostics, Mannheim, Germany) using SYBR Green (QuantiFast SYBR Green PCR Kit; Qiagen). Gene expression was analyzed in various pancreas samples; *GAPDH* was used as an internal reference control.

### 2.9 DNA methylation and expression in pancreatic cell lines

To define the *SST* methylation and expression in pancreatic cell lines, four tumor cell lines—AspC1, MiaPaCa2, T3M4, and QGP1—were used. The mycoplasma‐free cells were grown in Dulbecco's modified Eagle’s medium (Gibco, Paisley, UK) supplemented with 10% FBS and penicillin/streptomycin at 37 °C and 5% CO_2_ in a humidified incubator. Direct bisulfite sequencing and qPCR were performed to define the pattern of *SST* promoter methylation and gene expression levels, respectively.

### 2.10 DNA demethylation using 5‐aza‐2´‐deoxycytidine

MiaPaCa2 cells were grown and treated with 10 µm 5‐aza‐dC (Sigma‐Aldrich, Heidelberg, Germany) for 72 h. The medium was changed every 24 h in order to supply fresh drug to the cells. Treated and untreated control cells were harvested and total RNA was extracted. RT–qPCR was performed to document *SST* expression levels in both treated and untreated cells.

### 2.11 Validation of results using external datasets

For external validation, different DNA methylation and expression datasets from GEO and TCGA were explored. First, for validation of *SST* downregulation in PDAC, we applied three microarray expression datasets from GEO (Zhang *et al.*, 2012: accession number http://www.ncbi.nlm.nih.gov/geo/query/acc.cgi?acc=GSE62452; Yang *et al.*, 2016: http://www.ncbi.nlm.nih.gov/geo/query/acc.cgi?acc=GSE28735; and Pei *et al.*, 2009: http://www.ncbi.nlm.nih.gov/geo/query/acc.cgi?acc=GSE16515) containing data on 150 PDACs and 122 normal samples. For validation of *SST* DNA hypermethylation, we analyzed 184 tumor and 10 normal samples from the TCGA‐PAAD project and 167 tumor and 29 normal samples from GEO (Nones *et al.*, 2014: http://www.ncbi.nlm.nih.gov/geo/query/acc.cgi?acc=GSE49149). Since the Genotype‐Tissue Expression (GTEx) project data showed that *SST* was highly expressed in different parts of brain and gastrointestinal tissues (Mele *et al.*, 2015), we also investigated the expression and methylation of the *SST* gene across other gastrointestinal cancer entities in the TCGA database: tumors and normal esophagus (TCGA‐ESCA), stomach (TCGA‐STAD), colon (TCGA‐COAD) and rectum (TCGA‐READ) tissues. Subsequently, also data from various other tumor entities were looked at lung adenocarcinoma, breast invasive carcinoma, prostate adenocarcinoma, head neck squamous cell carcinoma, liver hepatocellular carcinoma, bladder carcinoma, and kidney renal clear cell carcinoma. These datasets are available for download at the UCSC Xena database (http://xena.ucsc.edu/).

### 2.12 Analysis of cell proliferation and migration

Cell viability and proliferation upon treatment with different concentrations of octreotide were assessed by resazurin assay (Sigma‐Aldrich) on the PDAC cell lines MiaPaCa‐2 and AsPC‐1. In summary, 4000 cells were seeded in each well of a 96‐well plate and grown for 24 h. Then, the cells were treated with 10, 20, and 40 μm of octreotide, respectively. After 96‐h drug treatment, the relative fluorescence was measured. Cell migration was assayed using an *in vitro* wound‐healing assay (Ibidi, Gräfelfing, Germany) according to the manufacturer’s protocol. Briefly, 30 000 MiaPaCa‐2 cells were seeded in each compartment of 2‐well inserts. After 24 h, the inserts were removed and the cells were cultured with 1% FBS medium in presence or absence of 5 μm octreotide. Cell migration was observed under the microscope after 24‐, 48‐, and 72‐h periods, and images were acquired. The NIH (Bethesda, MD, USA) image processing software imagej was used to quantify the cf area from different locations of three replicate experiments each.

### 2.13 Cell‐free DNA isolation and bisulfite conversion

Circulating cfDNA was extracted from 1 mL of plasma of PDAC patients and healthy individuals using the QIAamp MinElute ccfDNA Kit (Qiagen). In total, cfDNA was isolated from 30 PDAC samples [15 nonmetastatic (M0) and 15 metastatic (M1)] and 18 control samples from healthy donors. In order to avoid large DNA fragments, which originate from white blood cells and represent the main part of ctDNA contamination (Moss *et al.*, 2018; Mouliere *et al.*, 2018), size selection was performed using Agencourt Ampure XP beads (Beckman Coulter, Krefeld, Germany) according to the protocol recommended by the manufacturer. Bisulfite conversion was carried out on total cfDNA samples (EpiTect bisulfite kit; Qiagen) using the manufacture’s protocol for low concentration DNA samples.

### 2.14 MethyLight droplet digital PCR

The droplet digital PCR (ddPCR) method was employed for sensitive detection of aberrant methylation at target CpG sites of *SST* in cfDNA isolated from PDAC and normal plasma samples. The list of primer and Taqman probe sequences is provided in Table S3. All steps including droplet generation, thermal cycling, and droplet reading were performed according to the manufacturer’s protocols (Bio‐Rad). To evaluate the sensitivity of the primer/probe assay for detection of methylated alleles, initial experiments were performed using calibration DNA samples of 100%, 10%, 1%, 0.1%, 0.01%, and 0% methylation that were prepared by mixing control DNA of 100% and 0% methylation (EpiTect PCR Control DNA Set; Qiagen).

### 2.15 Statistical analysis

Statistical analyses were performed using graphpad prism 6 software (GraphPad Software Inc., San Diego, CA, USA). Results are presented as the mean and SD unless mentioned otherwise. Normal distribution of variables was computed using D'Agostino‐Pearson normality test. Comparison of a continuous variable in two or more than two groups with normal distribution was performed using parametric test (*t*‐test or ANOVA). If the variable was not normally distributed, a nonparametric test (Mann–Whitney or Kruskal–Wallis) was applied. All *P*‐values were two‐sided, and *P* < 0.05 was considered statistically significant. Survival analysis was performed on expression and methylation data by KM plotter (http://kmplot.com) and MethSurv (https://biit.cs.ut.ee/methsurv/) tools (Modhukur *et al.*, 2018; Nagy *et al.*, 2018). A Cox proportional hazards regression model was used to determine hazard ratios.

## 3 Results

### 3.1 Genomewide DNA methylation profiling identified multiple aberrantly methylated loci in PDAC tissue DNA

**Fig. 1:**
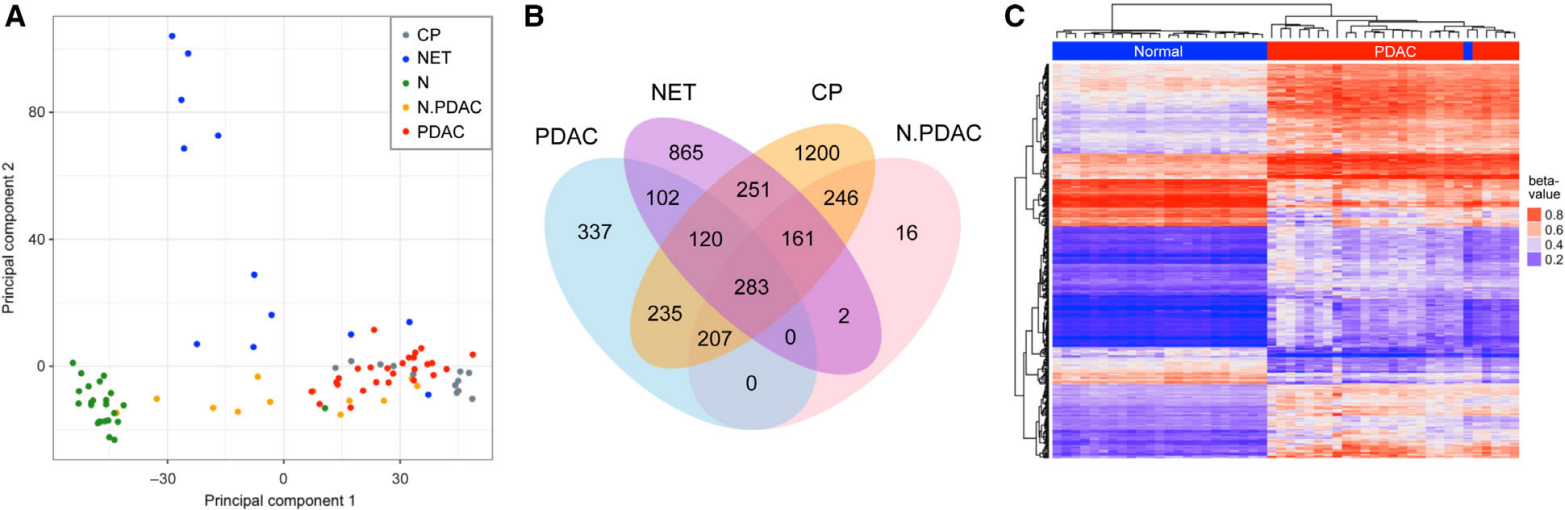
Differential DNA methylation in pancreatic tissue samples. (A**)** PCA of different sample types based on the information obtained by an analysis on the 450k microarray. The sample types are indicated. (B) Venn diagram showing the overlapping number of DMRs in four pancreatic sample types in comparison with normal samples. (C) Heatmap showing the DMRs in PDAC vs. normal pancreas (FDR < 0.01, Δβ > 15%). Each row stands for an individual promoter region based on the Infinium 450k array; each column represents a sample. Regions shaded in blue in the heatmap represent hypomethylated regions; regions shaded in red indicate hypermethylated regions.

After performing a genomewide analysis of DNA methylation by assaying more than 450 000 CpG sites across the genome with the 450k Illumina microarray, principal component analysis (PCA) of the data was performed to compare different samples based on the β‐values (degree of DNA methylation 0 < β < 1). In the analysis, a distinct cluster is formed by the normal tissue samples (Fig. 1A). The PDAC and CP samples were rather close with respect to methylation, which is different to a clustering according to transcriptional data, in which the CP samples fall in between normal and PDAC samples. This intermediate kind of pattern was actually exhibited by the methylation of the phenotypically normal‐looking peritumoral tissues (N.PDAC). The NET samples defined the second most principal component, indicating a particular methylation pattern that is not shared with any of the other sample groups. A Venn diagram shows which DMRs compared to normal tissues differ between or are shared among PDAC, NET, CP, and N.PDAC samples (Fig. 1B). Unsupervised hierarchical clustering was conducted using DMRs that were significantly differentially methylated between PDAC and normal pancreas tissues (Fig. 1C). The location of the CpGs that were differentially methylated in pancreatic tumors compared to normal tissues was annotated according to the UCSC classification of CGI (Dreszer *et al.*, 2012) (Fig. S2).

### 3.2 Selection of *SST* hypermethylation as candidate for further analysis

The genes associated with the top hypermethylated DMR candidates (622 genes, provided in Table S4) were used for functional enrichment analysis with Ingenuity IPA and ConsensusPathDB‐human predicting their relevance to cancer and gastrointestinal disease, besides other diseases and biofunctions (Fig. S3). Data from transcriptional profiling (Bauer *et al.*, 2018) showed that 2341 genes exhibit significant downregulation at the transcript level in PDAC compared to normal pancreas. The overlap between the two lists consists of 70 genes of both a higher degree of methylation and a concomitantly lower level of the respective transcript (Table S5). Based on the functional enrichment analysis, we focused on genes involved in important cancer processes such as proliferation, invasion, migration, cell death, and apoptosis signaling, thereby prioritizing the genes further. Furthermore, we looked for genes encoding proteins with known function in the gastrointestinal tract (Fig. S4).

**Fig. 2:**
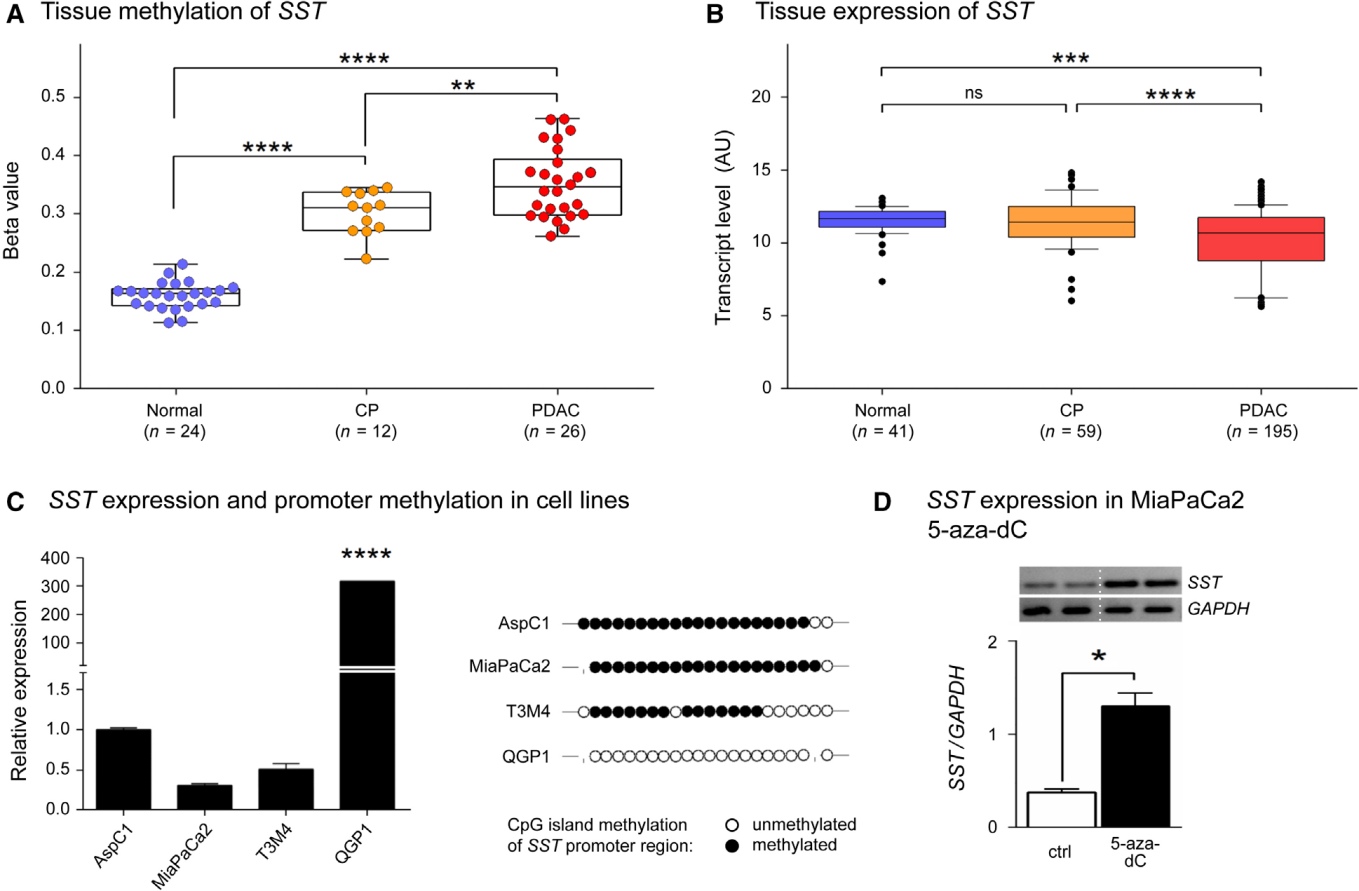
Correlation of *SST* methylation and expression. (A) The *SST* methylation level (mean of β‐values) in 26 PDAC and 12 CP tissues as well as 24 samples of healthy pancreas is shown. (B) Normalized *SST* gene expression data are presented that originate from the analysis of 195 PDAC, 59 CP, and 41 normal tissue samples. (C) *SST* methylation and expression in four cell lines as determined by direct bisulfite sequencing and RT–qPCR, respectively. Filled (black) circles correspond to methylated CpGs, open (white) circles to unmethylated CpGs; small vertical lines in place of a circle indicate missing information (e.g., caused by sequencing errors). All other bases are not shown in the graph. (D) Effect of treatment with 5‐aza‐dC, which causes complete DNA demethylation. Cell line MiaPaCa2 was grown in absence (ctrl) or presence of 5‐aza‐dC. The resulting variation in gene expression is shown as bands after gel electrophoresis of RT–PCR products as well as a quantification of the band intensities. Results are shown as mean values ± SD of the experiments. A two‐tailed unpaired Student’s *t*‐test was applied. ns: *P* > 0.05; *P ≤ 0.05; **P ≤ 0.01; ***P ≤ 0.001; ****P ≤ 0.0001.

Among the 70 genes, there were several with functions in either cell proliferation (*GPAM*, *KLB*, *NUPR1*, *PLA2G1B*, *REG1A*, *VIPR2,* and *SST*), cell migration and invasion (*FAM107A* and *SST*), apoptosis or cell death (*NUPR1*, *EEF1D*, *GPAM* and *SST*) as well as gastrointestinal function (*SLC9A4*, *AMY2A*, *CEL*, *CLPS*, *CTRB1*, *CTRB2*, *PNLIP*, *PNLIPRP2*, *PRSS1,* and *SST*). However, *SST* was the only gene that was involved in all categories; therefore, we selected it for further analysis. *SST* is a highly expressed gene in normal pancreas. It encodes for the hormone somatostatin, which was initially known as a regulator of growth hormone released from the anterior pituitary and has inhibitory effects on all known hormones of the gastrointestinal tract (Modlin *et al.*, 2010). In our analysis, the *SST* methylation and expression data showed a significant inverse difference in PDAC compared to the levels in normal samples (Fig. 2A,B). DNA methylation is higher while simultaneously *SST* expression is lower. Interestingly, in tissues from CP patients, expression of *SST* was not different compared to that in healthy tissues. To confirm that the *SST* gene is regulated through DNA methylation, we also looked at the gene’s methylation and expression in cell lines of PDAC and endocrine tumor. As expected, *SST* expression was significantly higher in the endocrine somatostatinoma cell line QGP1 compared to the three PDAC cell lines AspC1, MiaPaCa2, and T3M4 (Fig. 2C). DNA methylation analysis of the respective *SST* promoter revealed that all CpG sites were completely unmethylated in QGP1, which highly expresses *SST*, but is methylated in the other cell lines with very low *SST* expression (Fig. 2C). For one of the PDAC cell line—MiaPaCa2—we looked further at the change in *SST* expression upon treatment with 5‐aza‐dC, a compound that results in DNA demethylation. As expected, the *SST* expression increased (Fig. 2D) as a consequence of the treatment.

### 3.3 Validation of *SST* gene hypermethylation and downregulation in PDAC

**Fig. 3:**
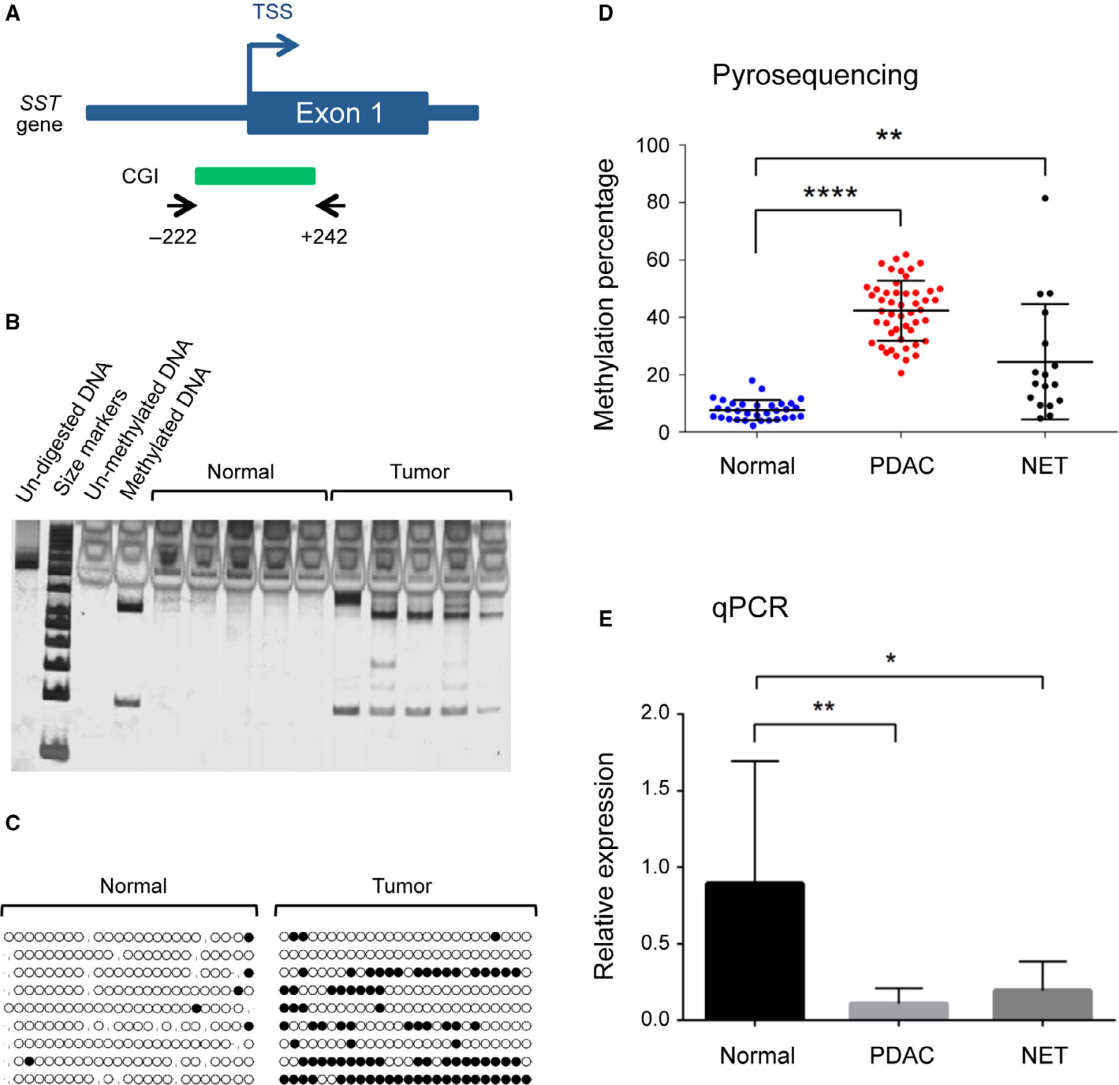
Validation of *SST* gene expression and methylation. (A) Schematic representation of the *SST* gene, the CGI, and the primer locations (arrows). (B) COBRA results of *SST* gene methylation. The CGI was treated with bisulfite, PCR‐amplified, and digested using *Bst*UI. All tumor samples exhibited some degree of digestion while none was detectable in the DNA from healthy controls (normal). (C) Representative results of bisulfite sequencing of the *SST* gene in PDAC tissues and samples from normal pancreas. Black and open circles represent methylated and unmethylated CpG sites, respectively. (D) Average methylation levels of four investigated CpG sites between healthy tissue (normal), PDACs, and NET samples using pyrosequencing. (E) Real‐time qPCR analysis of *SST* gene expression comparing normal tissues with PDAC and NET tumor samples. Results are shown as mean values ± SD of the experiments. A two‐tailed unpaired Student’s *t*‐test or ANOVA was used to evaluate the changes. *P ≤ 0.05; **P ≤ 0.01; ****P ≤ 0.0001.

The *SST* promoter contains a CGI, which covers the region upstream of the transcription starting site (TSS) and reaches into exon 1 (Fig. 3A). A 464‐bp region containing 26 CpG dinucleotides (location −222 to +242 relative to TSS) was studied by COBRA with samples already used on the 450k array. Normal control samples were not cut indicating a lack of DNA methylation; however, there was cleavage in the DNA isolated from tumor samples (Fig. 3B). Quantification by direct bisulfite sequencing of the amplified *SST* 464‐bp region showed that an average 37.2% of CpGs in tumor samples were methylated while only 2.2% methylation was observed in normal pancreas tissues (Fig. 3C). In addition, quantitative methylation results obtained by pyrosequencing were produced to validate further *SST* hypermethylation. Four CpG sites at positions +12, +23, +30, and +39 of the coding sequence of exon 1 were assessed. The four CpGs were studied in 49 PDAC, 17 NET, and 33 normal pancreas tissues. Of these samples, 26, 12, and 23, respectively, had already been used in the genomewide analysis (technical validation) while 23, 5, and 10 samples, respectively, were additional and independent ones (biological validation). Tumors exhibited a significant hypermethylation (Fig. 3D). Also, real‐time qPCR was performed for quantifying *SST* gene expression in normal and tumor tissue samples. There was a significant decrease in *SST* expression levels in PDACs and NETs compared to normal tissue (Fig. 3E). A Pearson’s correlation analysis of the association of *SST* methylation and the gene’s expression level yielded a significant (*P* = 0.001) negative correlation (*r* = −0.6641).

**Fig. 4:**
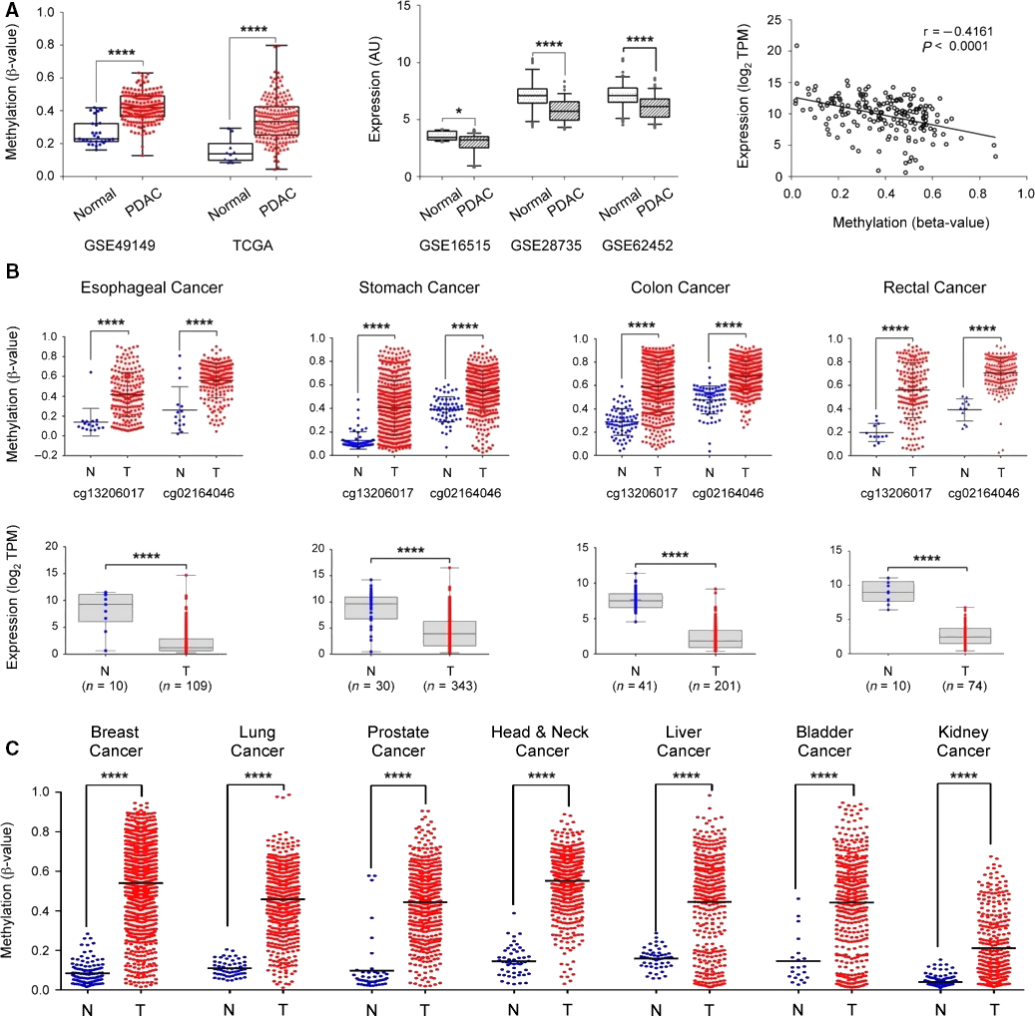
External validation of *SST* expression and methylation. (A) The *SST* gene methylation was looked at in two independent datasets about pancreatic cancer obtained from GEO and TCGA (left panel). The central panel shows the downregulation of *SST* gene expression in PDAC in three independent datasets from GEO. In the right panel, the Pearson correlation of DNA methylation and gene expression levels of the *SST* gene are shown (data from TCGA‐PAAD). (B) Investigation of *SST* gene methylation and expression in esophageal, stomach, colon, and rectal adenocarcinomas. The β‐values of two probes that were common to the 450k and 27k methylation arrays were applied for comparison. (C) *SST* gene methylation results are shown as in panel B but for breast, lung, prostate, head and neck, liver, bladder, and kidney cancer. TPM: transcripts per million; *P ≤ 0.05; ****P ≤ 0.0001.

For further confirmation, we also looked at methylation data available from the pancreatic cancer study (PAAD) within TCGA data repository and the http://www.ncbi.nlm.nih.gov/geo/query/acc.cgi?acc=GSE49149 dataset available in the GEO. Put together, they contain information about 351 pancreatic tumor and 39 normal tissues. For the analysis, the β‐values of the 10 Illumina probes were used that target the *SST* gene. There was significant hypermethylation in the tumors compared to normal tissues, confirming our findings (Fig. 4A). Moreover, for validation of *SST* downregulation, we used expression data from other, independent cohorts of pancreatic cancer available in GEO. This dataset contains expression profiles of 150 PDAC and 122 normal tissue samples. Congruent with our results, significant downregulation of the *SST* gene could be observed in PDAC tumors. Moreover, a Pearson’s correlation of the *SST* methylation profile with expression levels using methylation and expression data of pancreatic cancer in TCGA‐PAAD found a significant inverse correlation (*r* = −0.4161, *P* < 0.0001).

### 3.4 *SST* methylation as a pan‐cancer biomarker

Somatostatin exhibits inhibitory effects on all known hormones of the gastrointestinal tract (Modlin *et al.*, 2010). In order to learn how specific the variations in *SST* methylation and expression are to pancreatic cancer, we took advantage of TCGA expression and methylation data for an analysis of other gastrointestinal cancers. The corresponding data for tumors and normal ESCA, TCGA‐STAD, colon, and TCGA‐READ tissues were studied. As true for PDAC, *SST* methylation and expression in other gastrointestinal cancers showed highly significant hypermethylation and downregulation of expression (*P* < 0.0001; Fig. 4B). Subsequently, we extended the investigation to tumor entities beyond the gastrointestinal tract, for which appropriate data were available: TCGA methylation data from breast, lung, prostate, and head and neck cancer as well as liver, bladder and kidney tumors were analyzed. For all cancers, a comparison of tumor and healthy tissue samples revealed significant *SST* hypermethylation in tumor (Fig. 4C), implying that *SST* hypermethylation is a general and common feature in a broad range of tumors and could be utilized as a pan‐cancer biomarker.

### 3.5 Pharmacologic agonist of somatostatin reduced cell proliferation and migration

**Fig. 5:**
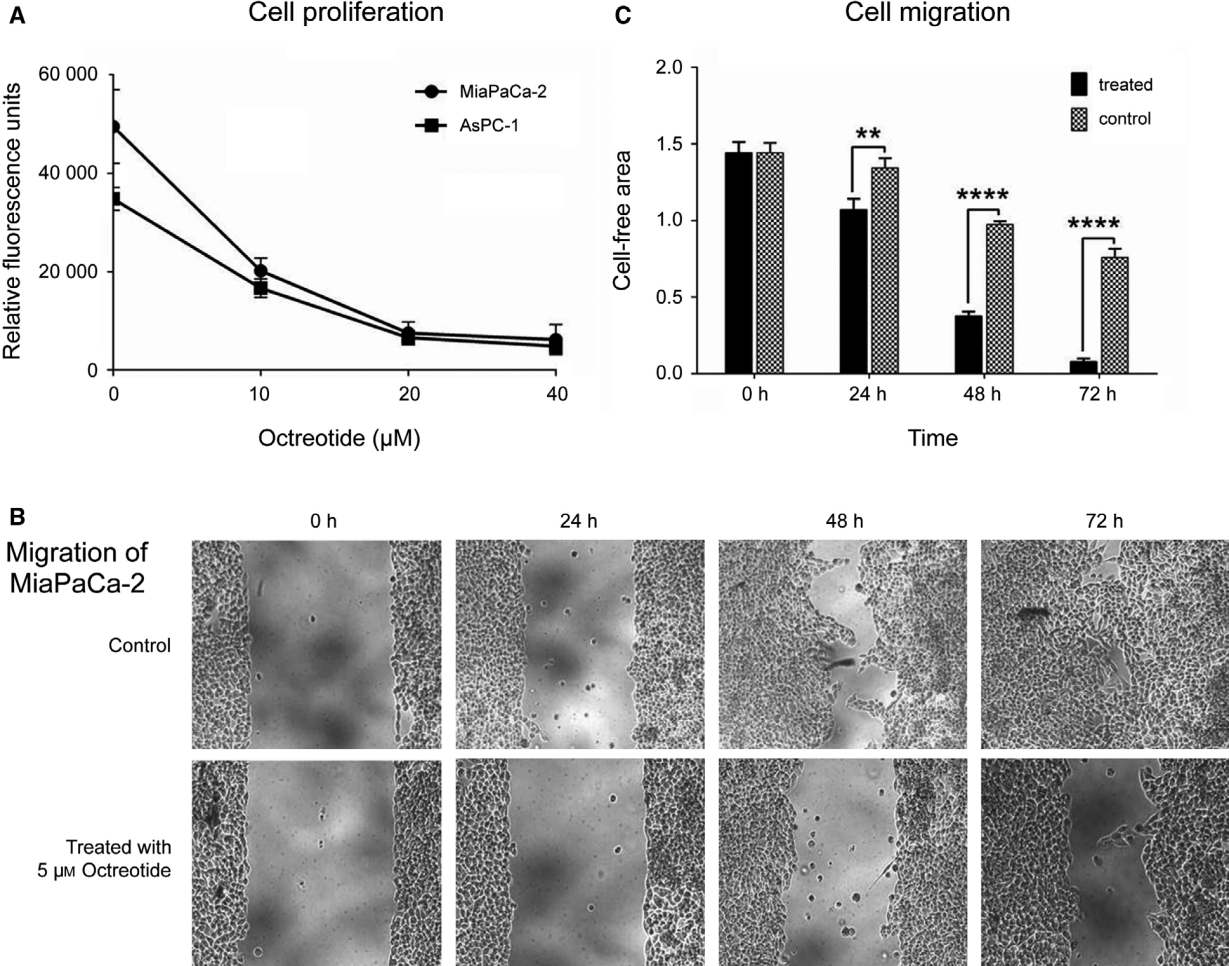
Inhibition of PDAC cell proliferation and migration in response to octreotide treatment. (A) Treatment of MiaPaCa‐2 and AsPC‐1 pancreatic cell lines with different concentration of the somatostatin agonist resulted in a significant decrease in cell growth (four biological replicates per experiment; two independent experiments) (B) Measurement of the inhibitory effect of octreotide treatment on migration of MiaPaCa‐2 pancreatic cancer cells in a wound‐healing assay. Typical results are shown. (C) Quantification of analyses, such as in panel B; each column represents the mean of three measurements (three biological replicates per experiment; two independent experiments). Results are shown as mean values ± SD of the experiments. A two‐tailed unpaired Student’s *t*‐test or ANOVA was used to evaluate the changes.

We determined the effect of the somatostatin agonist octreotide on cell proliferation and migration. Treatment with different octreotide concentrations resulted in an inhibitory effect on cell proliferation in the PDAC cell lines MiaPaCa‐2 and AsPC‐1 compared to untreated controls (Fig. 5A). Afterward, we studied whether octreotide had an effect on PDAC cell migration by assessing the motility of MiaPaCa‐2 cells in a wound‐healing assay in the presence of 5 μm octreotide. Octreotide treatment significantly suppressed the migration of MiaPaCa‐2 cells (Fig. 5B,C). These data confirmed that *SST* regulation modulates cancer‐related pathways involved in cell proliferation and migration.

### 3.6 Disease prognosis and diagnostic accuracy

**Fig. 6:**
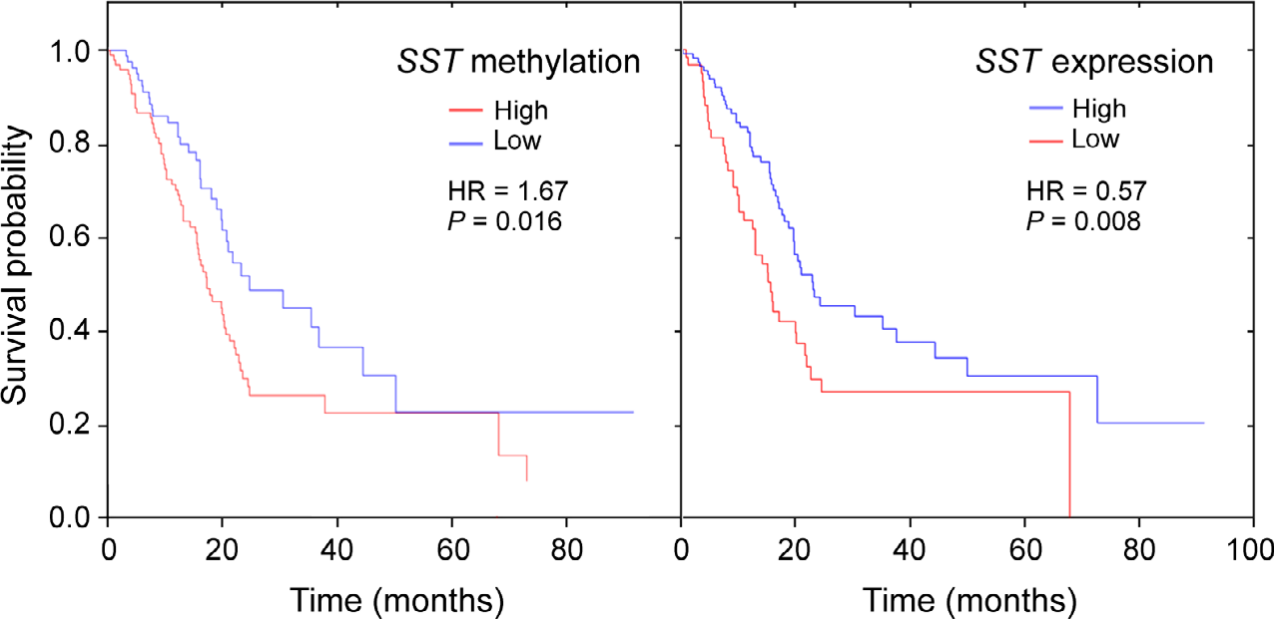
Prognostic performance of *SST* methylation and expression. Kaplan–Meier plots show the prognostic power of both methylation (left panel) and expression (right panel) of *SST*. HR stands for hazard ratio; also, the respective *P*‐value is given.

To evaluate whether there would be any prognostic value of *SST* promoter methylation, a univariate survival analysis regarding overall survival of PDAC patients was performed on our dataset using the median value as cutoff. The analysis revealed no statistically significant association between *SST* methylation and expression with the survival rate of PDAC patients. However, analyzing public datasets, which contain results from more samples (in total 184), did yield a prognostic value of *SST* methylation (*P* = 0.016 for cg02164046 from TCGA‐PAAD) and expression (*P* = 0.008 for TCGA‐PAAD) (Fig. 6). The Kaplan–Meier plots of *SST* methylation and expression exhibit very good congruence, although resulting from different datasets. Taking into account that low methylation causes high expression and vice versa, this confirms further the direct connection between the two.

For an evaluation of the diagnostic performance of *SST* methylation, a receiver operating characteristic (ROC) curve analysis was conducted using the average methylation degree of the four CpGs analyzed in the pyrosequencing (validation) experiment. Applying a threshold of 20% methylation, all PDAC tissue samples could be accurately discriminated from control tissue samples based solely on the degree of *SST* methylation. The area under the curve (AUC) value was 100% (*P* < 0.0001). The *SST* gene methylation could also differentiate NETs from healthy pancreas, although with a substantially lower AUC value of 86.3% (*P* < 0.001).

### 3.7 Sensitive detection of *SST* methylation from cfDNA by ddPCR

**Fig. 7:**
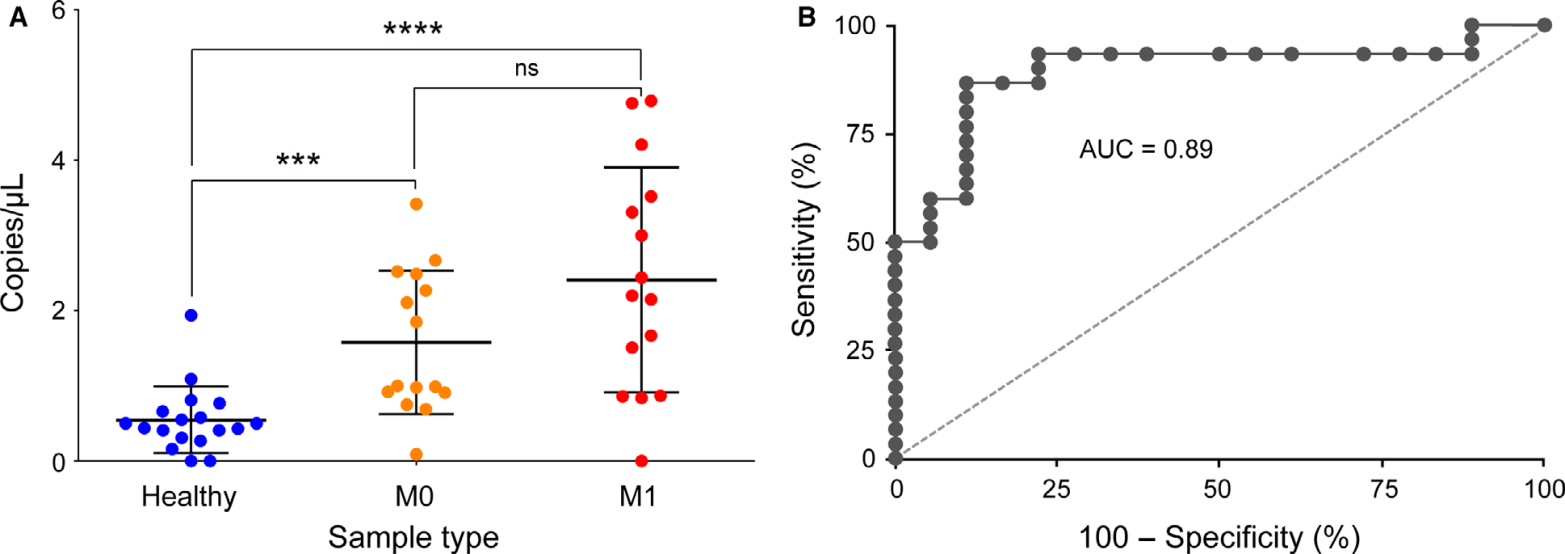
Results from analyzing cfDNA from plasma samples. ddPCR was used to study the abundance of the methylated *SST* allele in plasma samples of PDAC patients (M0: nonmetastatic; M1: metastatic) and healthy controls. ns: *P *> 0.05; ***P ≤ 0.001; ****P ≤ 0.0001.

For clinically applicable diagnosis, it would be advantageous, if a noninvasive process could be implemented. Therefore, we wondered, if tumor‐specific methylation may be detectable in circulating free (cf) DNA in the plasma of PDAC patients. ddPCR was applied for detection and quantification; the primer and probe sequences are provided in Table S3. The assay was found to be capable of detecting methylated *SST* from 100% down to 0.01% (methylated/ unmethylated *SST* allele) in a bulk of unmethylated DNA. For the actual analysis, cfDNA was isolated from plasma samples. ddPCR detected *SST* methylation in nearly all cfDNA samples ranging from 0.09 to 4.79 copies per μL. However, the number of copies per μL of *SST* methylation was significantly higher in PDAC (M1 and M0) than control samples (Fig. 7A). Also, there was a trend of more copies per µL in M1 than M0, although not at a level of statistical significance. With a threshold of 0.825 copies per μL, our *SST* methylation biomarker assay by ddPCR exhibited a sensitivity of 93.3% and a specificity of 88.9%. ROC analyses of the data from plasma samples yielded an AUC value of 89% (*P* < 0.0001; Fig. 7B).

## 4 Discussion

Using genomewide DNA methylation experiments, we showed that the *SST* gene is hypermethylated in pancreatic tumors. In addition, a significant inverse correlation between DNA methylation and gene expression was revealed. A most interesting result was the fact the *SST* hypermethylation and concomitant downregulation occurred in tumor DNA but not in material isolated from CP patients, which showed similar methylation variations but a different response at the transcript level. The combined effect is clearly tumor‐associated and not related to inflammation processes. The differences of PDAC and NET, most apparent in the PCA (Fig. 1), indicate that different CpGs are affected by methylation changes, which overall have the same consequence, however, with respect to downregulation of the *SST* gene expression.

While earlier data indicated that *SST* methylation may be a marker for PDAC, our results imply that it does not exhibit specificity for PDAC but could act as a marker useful for detecting many different tumors. *SST* gene expression may simultaneously discriminate tumor from inflammation (AUC = 0.74). Expression data on normal tissue samples from several projects, such as GTEx (Mele *et al.*, 2015), showed that somatostatin is expressed throughout the body, but at higher levels in the brain, pancreas, and across the various tissues of the gastrointestinal tract. Therefore, we explored the *SST* gene methylation and expression in other cancer entities available in the TCGA database (Tang *et al.*, 2017). Interestingly, the results showed that *SST* was significantly downregulated in the gastrointestinal tract malignancies of esophageal, stomach, colon, and rectal cancers. The methylation levels in tumor samples of the digestive system from TCGA were concomitantly significantly higher compared to normal tissue suggesting *SST* hypermethylation as a general mechanism of somatostatin downregulation in these types of tumors. In agreement with our results, there are reports on *SST* hypermethylation in gastrointestinal tract malignancies including esophageal (Jin *et al.*, 2008), stomach (Jackson *et al.*, 2011), and colorectal cancer (Mori *et al.*, 2006). It is interesting to note that gastrointestinal tumors exhibit a high expression of SST receptors, a fact that might represent a feedback mechanism following the decreased expression of somatostatin (Reubi *et al.*, 1994; Tang *et al.*, 1998; Zhao *et al.*, 2014).

To learn whether *SST* hypermethylation is specific to gastrointestinal tumors, we explored TCGA data of five additional major tumor entities beyond the gastrointestinal tract: lung, breast, prostate, head and neck, as well as liver cancer. In all the eventually 10 tumor types looked at, there were strong *SST* hypermethylation and downregulation of SST expression. This inverse correlation is not necessarily expected; transcription factors have been found that activate gene expression despite or rather because of a hypermethylated promoter (Wan *et al.*, 2017). Obviously, *SST* hypermethylation is a general and common process in a broad range of tumors, suggesting that somatostatin may function as a tumor suppressor.

The somatostatin receptors belong to the G protein‐coupled receptor superfamily, which recruits several downstream transduction signals such as adenylyl cyclase and calcium channels upon somatostatin binding. Through binding to high‐affinity G protein‐coupled somatostatin receptors (SSTR1 to 5) on the cell membrane, somatostatin plays different roles. It is a negative regulator of cell proliferation and migration, has cytostatic effects on tumor cells, and induces apoptosis through different pathways (Modlin *et al.*, 2010). Furthermore, somatostatin may also have antiangiogenic properties by inhibiting the production and release of angiogenic factors (Bocci *et al.*, 2007). Synthetic analogs of somatostatin have made possible advances in the treatment of NETs (Modlin *et al.*, 2010). Somatostatin and analogs thereof bind to different receptors and arrest cell growth using direct and indirect mechanisms (Susini and Buscail, 2006). It has also been reported that somatostatin analogs reduce hormone‐related morbidity and increase the time to progression in NET of the small intestine, which is the most common small intestinal malignancy (Stalberg *et al.*, 2016).

Somatostatin and dopamine have some similar structural and functional characteristics, and heterodimerization of SSTRs with dopamine receptors create a novel receptor with enhanced functional activity (Rocheville *et al.*, 2000). Interestingly, in a previous study, deregulation of dopamine receptor D2 (DRD2) was shown in PDAC tissues compared with nontumor tissues and DRD2 inhibition strongly affected PDAC metastasis (Jandaghi *et al.*, 2016). We also found that the somatostatin agonist octreotide has antiproliferative and antimigratory effects on PDAC cell lines, providing more evidence on its tumor suppressive roles in pancreatic cancer. In agreement with our results, a study showed that PDAC cells upregulate *SST* expression following demethylation by 5‐aza‐dC treatment (Gailhouste *et al.*, 2018), and the somatostatin analog octreotide decreases the PDAC cell growth, suggesting a possible therapeutic application of somatostatin analogs for combination therapy of PDAC.

Aberrant *SST* hypermethylation and expression was associated with the survival rate of PDAC patients based on TCGA data, implying its prognostic value for stratifying patients into high‐risk and low‐risk groups (HR = 1.67 and HR = 0.57, respectively). The result showed a low‐risk group of patients with higher *SST* mRNA levels or lower *SST* methylation score, had significantly longer survival time. So far, carbohydrate antigen (CA) 19‐9 remains the only approved biomarker also for response assessment but is limited by low sensitivity and specificity (Chang and Kundranda, 2017); therefore, further investigations are needed for the discovery of new markers for pancreatic cancer outcome prediction and management. Our result is in agreement with a previous study, which showed that PDAC patients survival was associated with hypermethylation of several individual genes including *SST* (HR = 1.63, *P* = 0.03) (Henriksen *et al.*, 2016). In this regard, Bydoun *et al. *(2018) reported a S100A10 mRNA and methylation status as predictive of pancreatic cancer survival where hypermethylation and lower expression were correlated with better survival. In another study, DNA methylation and altered expression of MET and ITGA2 was associated with patient survival and patients with coordinated hypomethylation and high expression of MET and ITGA2 strongly correlated with poor outcome (*P*‐value = 0.007 and 0.040, respectively) (Nones *et al.*, 2014).

With respect to diagnostics, the AUC value of *SST* methylation in tissue and plasma samples were 100% and 89%, respectively, demonstrating its high potential as a biomarker for cancer diagnosis. In comparison with the only available and approved serum biomarker for PDAC, CA 19‐9, our result shows a higher diagnostic sensitivity and specificity with about 93% vs. 80% and 89% vs. 86%, respectively (Hasan *et al.*, 2019; Zhang *et al.*, 2015). In addition, 10% of Caucasians lack the ability to produce CA‐19‐9. Although the difference between the PDAC M0 and M1 groups is not statistically significant (*P*‐value = 0.08), the mean level of methylated *SST* allele was higher in M1 compared to M0 (2.409 vs. 1.577 copies per µL). The increase supports the notion of the relevance of methylation for tumor progression.

The result of the gene’s methylation status in plasma‐derived cfDNA is in agreement with a previous study by Henriksen *et al. *(2017) which showed DNA methylation in cfDNA to be diagnostically relevant for pancreatic adenocarcinoma. The overall diagnostic performance of the selected 28‐gene promoter panel was demonstrated by an AUC value of 0.86. In a study by Kisiel *et al*. on tumor tissue and pancreatic juice using several DNA methylation biomarkers, *CD1D* was reported as the best marker that highly discriminated pancreatic cases from controls. Detection of *CD1D* methylation in pancreatic juice provided a better AUC compared to our blood biomarker (92% vs. 89%); however, collecting pancreatic juice is a more invasive procedure compared to blood collection. Another study identified *BNC1* and *ADAMTS1* hypermethylation as potential biomarker to detect early‐stage pancreatic cancer, with sensitivities of 79% and 48% and specificities of 89% and 92%, respectively. In our study, *SST* hypermethylation shows a higher sensitivity (93%) and similar specificity (89%).

From our tissue data, one would expect that *SST* methylation will not be usable as a marker of pancreatic cancer in particular. Instead, it is likely that *SST* hypermethylation has the potential as a blood‐based pan‐cancer biomarker for a broad range of tumors for initial screening in order to stratify individuals into high‐risk and low‐risk groups. The high‐risk group could then be further evaluated by other means, such as CT imaging and/or endoscopic screening. In combination with existing biomarkers such as CA.19‐9 or KRAS mutations, the *SST* methylation should improve the diagnostic accuracy. Apart from its potential for noninvasive diagnosis, *SST* hypermethylation and its concomitant downregulation may lead to increased cell proliferation and migration. This supports a possible therapeutic application of somatostatin analogs in combination therapies.

## 5 Conclusion

Our results show that *SST* gene hypermethylation and simultaneous downregulation of expression occurs in pancreatic cancer and a broad range of other tumor entities, acting as a pan‐cancer molecular biomarker. Furthermore, *SST* hypermethylation and expression have prognostic value and are associated with the survival rate of PDAC patients. In addition, the methylation changes could also be found in liquid biopsy samples of PDAC patients, holding promise for a noninvasive diagnostic use.

## Conflict of interest

The authors declare that they have no competing interests.

### Author contributions

MM, ASB, and JDH designed the study. NAG, OS, and TH collected the samples and related clinical information. MM performed most of the experiments and repository data collection. YW did the bioinformatics analyses of the genomewide methylation data. NAG, OS, and SK performed tissue cryosectioning, DNA/RNA isolations, and material characterization. FH and EAM designed and executed the pyrosequencing experiments. TH, MM, ASB, and JDH interpreted the results and wrote the manuscript. All authors read and revised the manuscript critically and approved the final version.

## Ethics approval

The study was approved by the local ethics committee at Heidelberg University.

## Supporting information


**Fig. S1** **.** Graphical overview of the analysis processes and sample numbers used in the study.
**Fig. S2** **.** Pie charts are illustrating the proportions of probes with significant methylation differences in PDAC vs. normal tissue according to the UCSC classification of functional regions.
**Fig. S3** **.** Result of a functional enrichment analysis of genes associated with genomic regions that were found to be significantly hypermethylated.
**Fig. S4** **.** Schematic flow chart on the gene selection process.

**Table S1.** Demographic and clinical characteristics of the patients in this study.
**Table S3.** Sequences of the primers used in this study.
**Table S4.** Hypermethylated genes.
**Table S5.** Hypermethylated and down‐regulated genes.

**Table S2.** List of differentially methylated regions (DMRs).

## Data Availability

The HumanMethylation450 and transcriptional profiling data are available in the ArrayExpress (European Bioinformatics Institute) under accession numbers E‐MTAB‐3855 and E‐MTAB‐1791, respectively. The TCGA data analyzed in the current study were obtained from UCSC Xena Portal (http://xena.ucsc.edu). They are also available at the NCI Genomic Data Commons Data Portal (https://portal.gdc.cancer.gov/). The NCBI GEO data are available at http://www.ncbi.nlm.nih.gov/geo/.
