# Supplementary material for: SST gene hypermethylation acts as a pan‐cancer marker for pancreatic ductal adenocarcinoma and multiple other tumors: toward its use for blood‐based diagnosis
Source: Mol Oncol. 2020 Apr 14;14(6):1252–67. doi: 10.1002/1878-0261.12684 (PMC7266283; doi:10.1002/1878-0261.12684)
Supplement: Supplementary file 1 — Fig. S1 . Graphical overview of the analysis processes and sample numbers used in the study. Fig. S2 . Pie charts are illustrating the proportions of probes with significant methylation differences in PDAC vs. normal tissue according to the UCSC classification of functional regions. Fig. S3 . Result of a functional enrichment analysis of genes associated with genomic regions that were found to be significantly hypermethylated. Fig. S4 . Schematic flow chart on the gene selection process. [file MOL2-14-1252-s001.docx]

**SUPPLEMENTARY FIGURES**

**
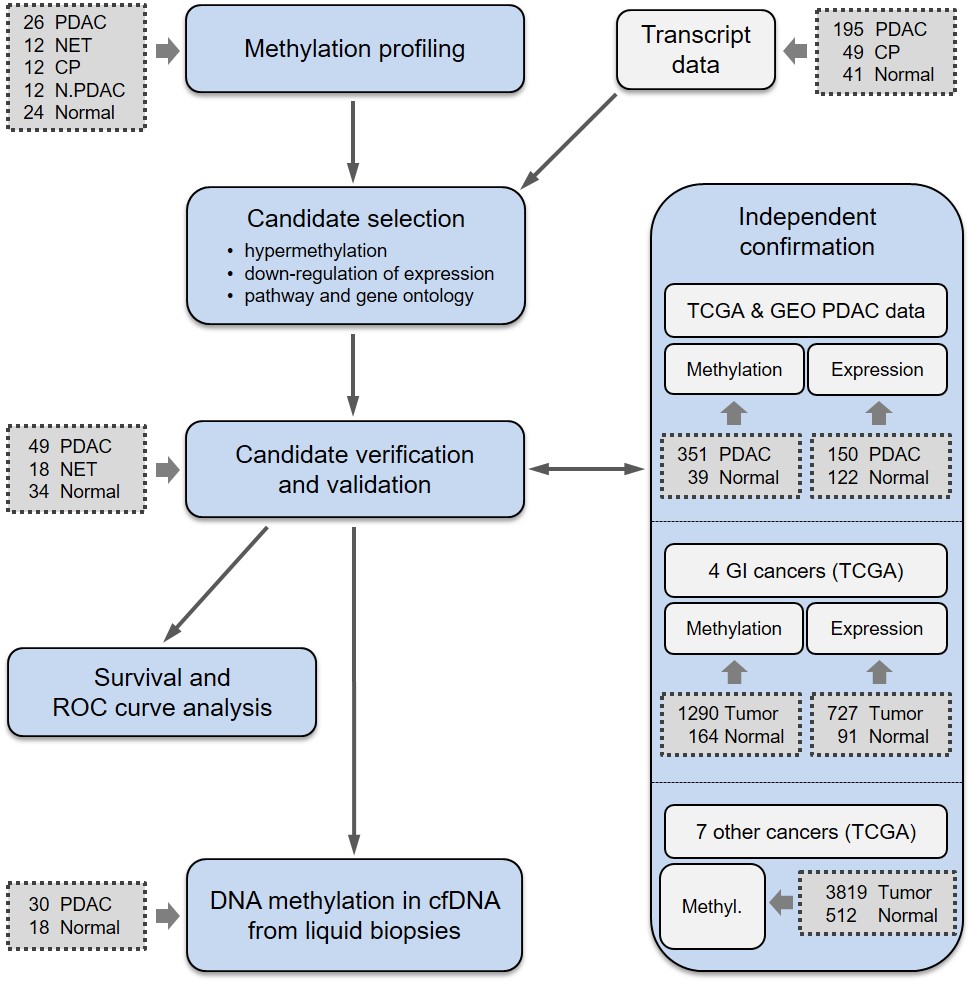
**

**Suppl. Figure 1.** Graphical overview of the analysis processes and sample numbers used in the study.


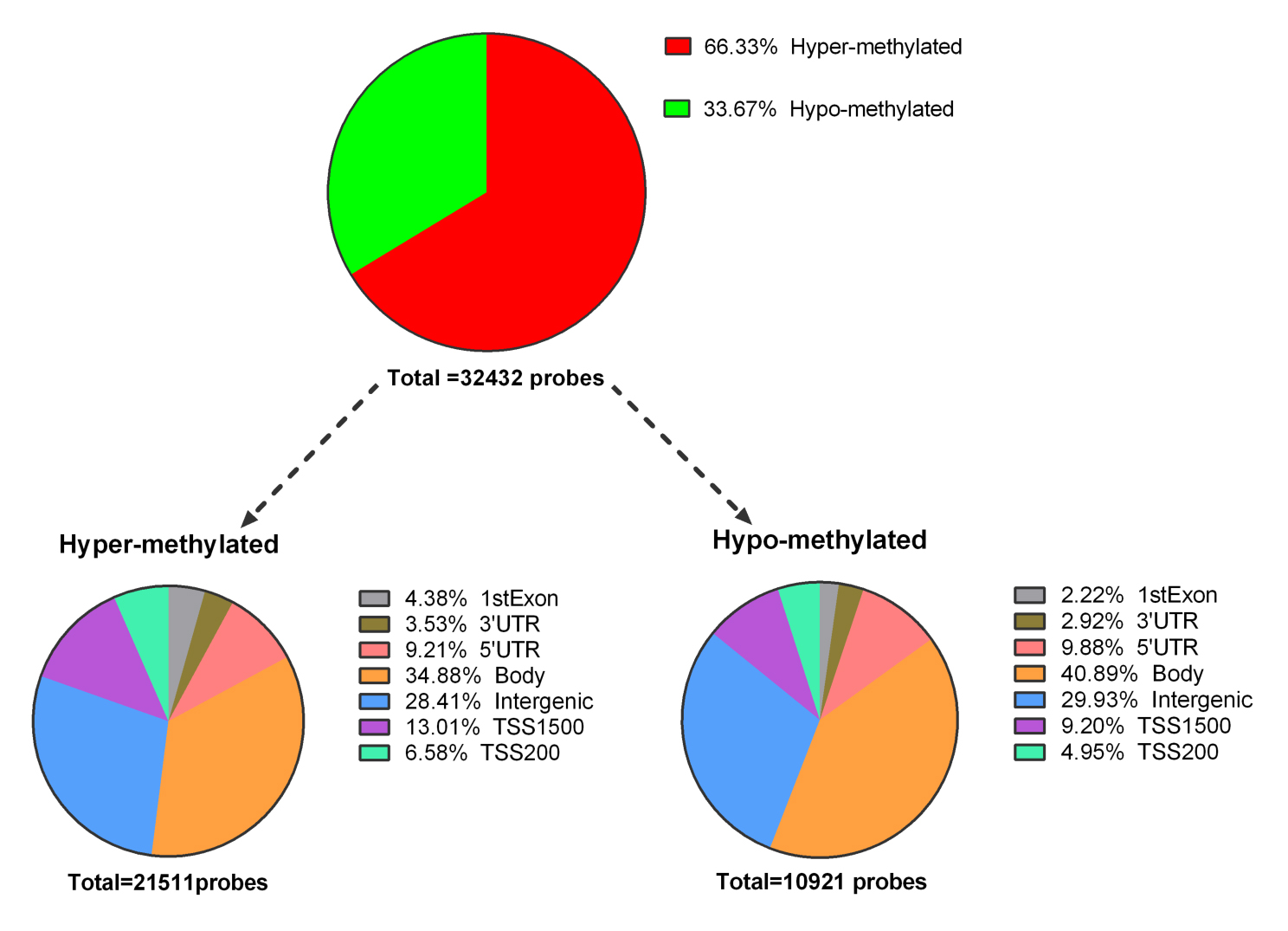


**Suppl. Figure 2.** Pie charts are illustrating the proportions of probes with significant methylation differences in PDAC versus normal tissue according to the UCSC classification of functional regions.


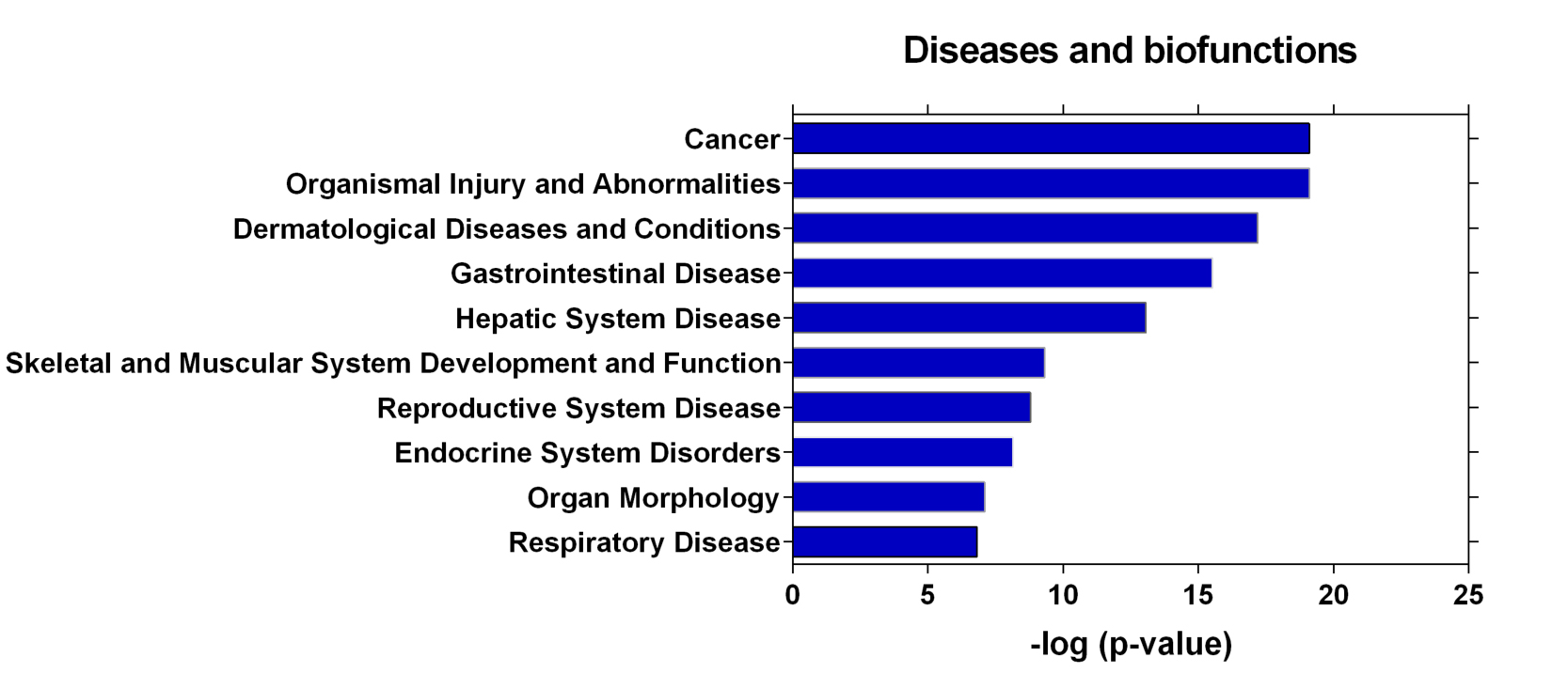


**Suppl. Figure 3.** Result of a functional enrichment analysis of genes associated with genomic regions that were found to be significantly hypermethylated.


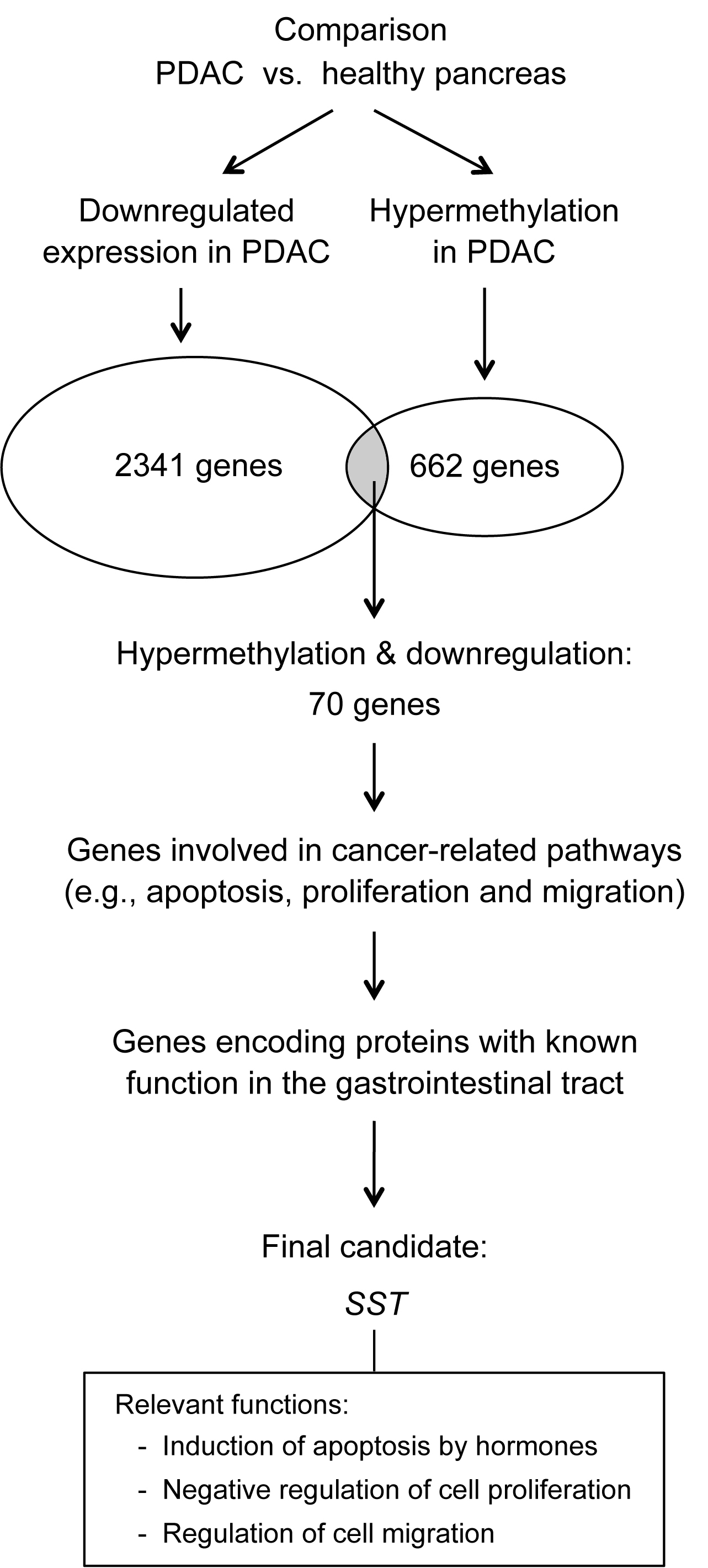


**Suppl. Figure 4.** Schematic flow chart on the gene selection process. In PDAC, 2341 genes exhibited significant down-regulation at the transcript level compared to normal pancreas and 662 genes showed hypermethylation of the promoter. Seventy genes were found in both lists. Based on functional information, we focused on genes involved in important cancer processes. Genes were then prioritized further by looking for genes encoding proteins with a known function in the gastrointestinal tract.
