# Supplementary material for: SST gene hypermethylation acts as a pan‐cancer marker for pancreatic ductal adenocarcinoma and multiple other tumors: toward its use for blood‐based diagnosis
Source: Mol Oncol. 2020 Apr 14;14(6):1252–67. doi: 10.1002/1878-0261.12684 (PMC7266283; doi:10.1002/1878-0261.12684)
Supplement: Supplementary file 2 — Table S1. Demographic and clinical characteristics of the patients in this study. Table S3. Sequences of the primers used in this study. Table S4. Hypermethylated genes. Table S5. Hypermethylated and down‐regulated genes. [file MOL2-14-1252-s002.docx]

**Supplementary Table 1.** Demographic and clinical characteristics of the patients in this study.

| **Sample type** | **PDAC** | **pNEN** |
| --- | --- | --- |
| **Total number** | 49 | 18 |
| **Gender** |  |  |
| Male (number and %) | 31 (63%) | 13 (72%) |
| Female (number and %) | 18 (37%) | 5 (28%) |
| **Mean age (range)** | 64 (44-81) | 56 (30-73) |
| **Stage** |  |  |
| I | 1 | - |
| II | 34 | 7 |
| III | 6 | 8 |
| IV | 7 | 2 |
| NA | 1 | 1 |
| **Grade** |  |  |
| G1/Gr1 | 1 | 6 |
| G2/Gr2 | 29 | 8 |
| G3/Gr3 | 16 | 4 |
| GX | 2 | - |
| NA | 1 | - |
| **Mean percentage tumor cells (range)** | 30 (5-70) | 75 (5-95) |
| **Survival time, month (range)** | 24.8 (2.9-147.7) | 43.4 (0.3-121.7) |

**Supplementary Table 3.** Sequences of the primers used in this study.

| **Experiment** | **Primer name** | **Sequences (5´-3´)** | **Amplicon length** |
| --- | --- | --- | --- |
| COBRA | *SST*-F | tagtattgagtgaaaataaaagattgtata | 464 bp |
|  | *SST*-R | aaccaaaaacttctacaaaaactaac |  |
| qPCR | *SST*-F | acccaaccagacggagaatga | 108 bp |
|  | *SST*-R | gccgggtttgagttagcaga |  |
|  | *GAPDH*-F | aaggtgaaggtcggagtcaac | 102 bp |
|  | *GAPDH*-R | ggggtcattgatggcaacaata |  |
| Pyrosequencing | *SST*-F | aatagagggagaaggttgagagtata | 253 bp |
|  | *SST*-R | bio-acaacaaccaaaaacttctacaa |  |
|  | *SST*-S | ggagaaggttgagagtatataag |  |
| ddPCR | *SST*-F | gagacggttgagagtatataagtc | 86 bp |
|  | *SST*-R | aatcaaactctaaacgcgaatca |  |
|  | *SST*-P | FAM-gataactccgaacctcgctc-BHQ1 |  |
|  | C-less-C1-F | ttgtatgtatgtgagtgtgggagagaga | 69 bp |
|  | C-less-C1-R | tttcttccaccccttctcttcc |  |
|  | C-less-C1-P | HEX-cctccccctctaactctat-BHQ2 |  |

F: forward primer; R: reverse primer; S: pyrosequencing primer; P: probe oligonucleotide used for detection of ddPCR-products; bio: biotinylation; FAM, HEX: reporter fluorophors; BHQ1, BHQ2: quencher molecules; C-less-C1: primers for genomic sequence that does not contain cytosine and is used for normalization.

**Supplementary Table 4.** Hypermethylated genes.

| *AJAP1* |
| --- |
| *ABCA8* |
| *ABCB10P4* |
| *ABCC11* |
| *ABCC9* |
| *ACACB* |
| *ACAN* |
| *ACTA1* |
| *ACTL6B* |
| *ACY3* |
| *ADAM12* |
| *ADAMTS16* |
| *ADAMTS2* |
| *ADAMTS20* |
| *ADAMTSL3* |
| *ADCY8* |
| *ADH1A* |
| *ADH6* |
| *AGAP2-AS1* |
| *AGMO* |
| *AK3* |
| *ALDH8A1* |
| *ALDOB* |
| *AMPH* |
| *AMY2A* |
| *ANKRD22* |
| *ANKS4B* |
| *AOX3P* |
| *APCDD1L-AS1* |
| *APOBEC2* |
| *AQP12A* |
| *AQP7P1* |
| *AQP8* |
| *ARHGDIG* |
| *ARHGEF38* |
| *ARL5C* |
| *ARPC3P1* |
| *ASB16* |
| *ASCL5* |
| *ATP2C1* |
| *ATP5A1P3* |
| *ATP6V1G3* |
| *BANF1P1* |
| *BANF2* |
| *BARHL2* |
| *BCAT1* |
| *BCL2L15* |
| *BHLHE23* |
| *BOLL* |
| *BRINP1* |
| *BSPRY* |
| *BTBD18* |
| *C10ORF129* |
| *C10ORF53* |
| *C10ORF71* |
| *C10ORF71-AS1* |
| *C11ORF83* |
| *C11ORF87* |
| *C11ORF94* |
| *C12ORF42* |
| *C12ORF74* |
| *C15ORF26* |
| *C17ORF104* |
| *C1ORF210* |
| *C1QTNF3* |
| *C22ORF42* |
| *C3ORF62* |
| *C4BPB* |
| *C4ORF50* |
| *C5* |
| *C5ORF38* |
| *C8A* |
| *C8ORF34* |
| *C9ORF117* |
| *C9ORF139* |
| *C9ORF152* |
| *CABLES1* |
| *CACNA1B* |
| *CACNB2* |
| *CACNG7* |
| *CACNG8* |
| *CAMK2A* |
| *CAPSL* |
| *CBLN4* |
| *CCDC19* |
| *CCNA1* |
| *CD36* |
| *CDH18* |
| *CDH20* |
| *CDH4* |
| *CDK5RAP3* |
| *CDRT15L2* |
| *CEL* |
| *CELA2A* |
| *CELA3A* |
| *CELA3B* |
| *CEP164* |
| *CERS4* |
| *CFL1P7* |
| *CHL1-AS2* |
| *CHODL-AS1* |
| *CIRBP-AS1* |
| *CKMT2* |
| *CLCA1* |
| *CLCNKB* |
| *CLDN8* |
| *CLIP3* |
| *CLPS* |
| *CNEP1R1* |
| *CNKSR1* |
| *CNOT7P2* |
| *CNTN4* |
| *CNTN6* |
| *CNTNAP5* |
| *COL18A1-AS1* |
| *COX11P1* |
| *CPA1* |
| *CPA2* |
| *CPA4* |
| *CPSF4L* |
| *CPXM1* |
| *CRMP1* |
| *CSMD1* |
| *CTNND1* |
| *CTRB1* |
| *CTRB2* |
| *CTRC* |
| *CTRL* |
| *CTSLP3* |
| *CTSLP6* |
| *CTXN3* |
| *CXCL17* |
| *CYP11A1* |
| *CYP3A43* |
| *CYP4B1* |
| *CYP8B1* |
| *CYYR1* |
| *DBX1* |
| *DCK* |
| *DEFB132* |
| *DGKI* |
| *DIP2A-IT1* |
| *DLEU7* |
| *DNALI1* |
| *DNMBP-AS1* |
| *DNMT3L* |
| *DOC2B* |
| *DPP6* |
| *DPYS* |
| *DRD5* |
| *DRD5P1* |
| *ECEL1P2* |
| *EDAR* |
| *EEF1D* |
| *EFCAB4A* |
| *EGLN1* |
| *EHF* |
| *ELAVL4* |
| *ELL2P1* |
| *ELP3* |
| *ELTD1* |
| *EMB* |
| *EPOR* |
| *ERICH1* |
| *ERP27* |
| *EVA1A* |
| *EVX1* |
| *EXOC3L2* |
| *EYA4* |
| *F11* |
| *FABP9* |
| *FAM107A* |
| *FAM129B* |
| *FAM135B* |
| *FAM154A* |
| *FAM169B* |
| *FAM177A1* |
| *FAM184A* |
| *FAM196B* |
| *FAM19A5* |
| *FAM60A* |
| *FBXL21* |
| *FBXL7* |
| *FBXW12* |
| *FERD3L* |
| *FGD5P1* |
| *FITM1* |
| *FOXB2* |
| *FOXC2* |
| *FOXI2* |
| *FOXO1B* |
| *FRMD4B* |
| *FSHR* |
| *FZD10* |
| *FZD10-AS1* |
| *GABRG1* |
| *GABRG3* |
| *GALNT13* |
| *GALNT9* |
| *GALR1* |
| *GBX2* |
| *GCC1* |
| *GFRA2* |
| *GGCT* |
| *GINS2* |
| *GLRA1* |
| *GP2* |
| *GPAM* |
| *GPHA2* |
| *GPR26* |
| *GPR56* |
| *GPT* |
| *GRAMD1B* |
| *GRB7* |
| *GRIA4* |
| *GRIK3* |
| *GRM5* |
| *GSG1L* |
| *GSTA5* |
| *GSTA7P* |
| *GUCA1C* |
| *HDDC2* |
| *HKDC1* |
| *HMGCLL1* |
| *HMGCS2* |
| *HNF1A* |
| *HNF4A* |
| *HOTAIRM1* |
| *HOXA10-AS* |
| *HOXA3* |
| *HOXA4* |
| *HOXA5* |
| *HOXB3* |
| *HOXB6* |
| *HOXB7* |
| *HOXC13-AS* |
| *HOXD-AS1* |
| *HOXD10* |
| *HOXD12* |
| *HOXD3* |
| *HOXD8* |
| *HOXD9* |
| *HS3ST2* |
| *HYPK* |
| *IL21-AS1* |
| *IL22RA1* |
| *INPP5J* |
| *INSL6* |
| *IQCJ* |
| *IQCJ-SCHIP1* |
| *IRGC* |
| *IRX1* |
| *IRX4* |
| *IRX6* |
| *ITGA8* |
| *ITIH2* |
| *KCNA3* |
| *KCNA4* |
| *KCNA5* |
| *KCNIP2* |
| *KCNIP4* |
| *KCNJ16* |
| *KHDRBS2* |
| *KIAA1211* |
| *KIRREL2* |
| *KLB* |
| *KRT38* |
| *KRT39* |
| *KRT86* |
| *KRT8P41* |
| *KRTAP4-8* |
| *LAMA1* |
| *LARP1* |
| *LCN2* |
| *LEP* |
| *LETM1P2* |
| *LHCGR* |
| *LHFPL4* |
| *LINC00111* |
| *LINC00315* |
| *LINC00403* |
| *LINC00404* |
| *LINC00445* |
| *LINC00485* |
| *LINC00577* |
| *LINC00617* |
| *LINC00707* |
| *LINC00880* |
| *LINC00922* |
| *LINC00940* |
| *LINC01091* |
| *LINC01105* |
| *LMO3* |
| *LNX1-AS2* |
| *LOXHD1* |
| *LPAL2* |
| *LRFN3* |
| *LRRC3B* |
| *LSM12* |
| *MACC1* |
| *MAGI1-IT1* |
| *MARVELD2* |
| *MAT1A* |
| *MATN4* |
| *MDGA2* |
| *MED15P3* |
| *MEP1A* |
| *METTL11B* |
| *METTL7AP1* |
| *MIR124-2* |
| *MIR124-3* |
| *MIR1282* |
| *MIR129-2* |
| *MIR190B* |
| *MIR194-1* |
| *MIR196A1* |
| *MIR2113* |
| *MIR215* |
| *MIR3174* |
| *MIR335* |
| *MIR3622A;*  *MIR3622B* |
| *MIR3667* |
| *MIR383* |
| *MIR4269* |
| *MIR4632* |
| *MIR4647* |
| *MIR4761* |
| *MIR548J* |
| *MIR5584* |
| *MIR5687* |
| *MIR5699* |
| *MIR575* |
| *MIR587* |
| *MIR629* |
| *MIR641* |
| *MIR759* |
| *MIR802* |
| *MS4A8* |
| *MTFR1L* |
| *MTMR7* |
| *MTNR1B* |
| *MYH14* |
| *MYLK2* |
| *MYOM1* |
| *MYT1L-AS1* |
| *NBL1* |
| *NBPF16* |
| *NEFH* |
| *NETO1* |
| *NKX1-1* |
| *NKX2-1-AS1* |
| *NKX2-6* |
| *NOX3* |
| *NPAS4* |
| *NPTX2* |
| *NPY5R* |
| *NR0B2* |
| *NUPR1* |
| *NXNL1* |
| *OLIG2* |
| *OR13J1* |
| *OR1G1* |
| *OR2A1-AS1* |
| *OR2L3* |
| *OR2S2* |
| *OR2T34* |
| *OR2W1* |
| *OR4S2* |
| *OR5AK2* |
| *OR6K3* |
| *OR7G1* |
| *OR8D1* |
| *ORM1* |
| *OTX2-AS1* |
| *PAH* |
| *PALMD* |
| *PAQR6* |
| *PAQR7* |
| *PAX3* |
| *PCAT7* |
| *PCDH10* |
| *PCDH8* |
| *PCDHA3* |
| *PCDHB15* |
| *PCDHB3* |
| *PCDHGA12* |
| *PCDHGA7* |
| *PCDHGB4* |
| *PCDHGB6* |
| *PCDHGC4* |
| *PDE4B* |
| *PDIA2* |
| *PDP2* |
| *PDZRN3* |
| *PDZRN4* |
| *PENK* |
| *PEX19* |
| *PEX5L* |
| *PGAM1P11* |
| *PHKG1* |
| *PHYHD1* |
| *PIAS2* |
| *PKD1L2* |
| *PLA2G1B* |
| *PLG* |
| *PLIN1* |
| *PNLIP* |
| *PNLIPRP1* |
| *PNLIPRP2* |
| *POLD4* |
| *POLR3G* |
| *POU3F3* |
| *PPIAP28* |
| *PPIAP31* |
| *PRAC2* |
| *PRB2* |
| *PRDM14* |
| *PRKCB* |
| *PRKCD* |
| *PROKR2* |
| *PROX1* |
| *PRPH* |
| *PRR15L* |
| *PRR26* |
| *PRRX1* |
| *PRSS1* |
| *PRSS8* |
| *PSMA2P3* |
| *PTPRN* |
| *PVRL4* |
| *PXDN* |
| *RAB26* |
| *RARRES2P6* |
| *RBBP8NL* |
| *RBFOX1* |
| *RBM47* |
| *REG1A* |
| *REG1P* |
| *RHOQP2* |
| *RHOQP3* |
| *RILP* |
| *RIMKLB* |
| *RN7SL650P* |
| *RNA5SP137* |
| *RNA5SP179* |
| *RNA5SP40* |
| *RNA5SP440* |
| *RNA5SP90* |
| *RNF128* |
| *RNF186* |
| *RNU1-149P* |
| *RNU4-63P* |
| *RNU6-1084P* |
| *RNU6-170P* |
| *RNU6-173P* |
| *RNU6-292P* |
| *RNU6-930P* |
| *RNU6-960P* |
| *RNU7-122P* |
| *RNU7-156P* |
| *RNY1P1* |
| *ROPN1* |
| *ROPN1B* |
| *RORC* |
| *RPL7P49* |
| *RPL7P51* |
| *RYR2* |
| *SAA3P* |
| *SALL1* |
| *SALL3* |
| *SAMD9L* |
| *SAPCD1-AS1* |
| *SBK2* |
| *SCARNA12* |
| *SCHIP1* |
| *SDCBP2* |
| *SDR16C6P* |
| *SERTM1* |
| *SFRP1* |
| *SGK2* |
| *SIX6* |
| *SLA* |
| *SLC11A2* |
| *SLC13A1* |
| *SLC15A2* |
| *SLC17A1* |
| *SLC18A3* |
| *SLC22A31* |
| *SLC24A4* |
| *SLC25A23* |
| *SLC27A6* |
| *SLC32A1* |
| *SLC35D2* |
| *SLC35F4* |
| *SLC5A10* |
| *SLC6A11* |
| *SLC6A15* |
| *SLC6A2* |
| *SLC6A3* |
| *SLC7A14* |
| *SLC9A4* |
| *SMAD3* |
| *SMIM17* |
| *SMIM3* |
| *SMIM6* |
| *SNORA23* |
| *SNORA55* |
| *SNORD19* |
| *SNORD71* |
| *SNORD83A* |
| *SNORD94* |
| *SORCS1* |
| *SORCS3* |
| *SOX1* |
| *SOX11* |
| *SOX17* |
| *SPAG6* |
| *SPHKAP* |
| *SPON1* |
| *SPSB3* |
| *SRD5A2* |
| *SRRM4* |
| *SST* |
| *SSTR3* |
| *SSTR4* |
| *SSTR5-AS1* |
| *ST6GAL2* |
| *STX19* |
| *SYN3* |
| *SYNDIG1L* |
| *SYT10* |
| *TAC3* |
| *TBC1D19* |
| *TBX20* |
| *TCEB1P18* |
| *TCERG1L* |
| *TCF15* |
| *TCP11* |
| *TDGF1* |
| *TERT* |
| *TFAP2E* |
| *TINAG* |
| *TLX3* |
| *TMEM108* |
| *TMEM125* |
| *TMEM132C* |
| *TMEM132D* |
| *TMEM179* |
| *TMEM190* |
| *TMEM72* |
| *TNRC6C-AS1* |
| *TPM4P1* |
| *TPTEP1* |
| *TRAJ58* |
| *TRAJ59* |
| *TRAJ60* |
| *TRBV29-1* |
| *TRBVB* |
| *TREH* |
| *TRH* |
| *TRIM40* |
| *TRIM58* |
| *TRIML2* |
| *TRPC7-AS1* |
| *TRPM2* |
| *TSACC* |
| *TSPAN31* |
| *TWIST1* |
| *TXNDC9* |
| *TXNP4* |
| *UCHL1* |
| *UCHL1-AS1* |
| *UGT1A2P* |
| *UGT2A3* |
| *UNC5D* |
| *UTF1* |
| *VAT1L* |
| *VAX1* |
| *VIPR1-AS1* |
| *VIPR2* |
| *VSIG10L* |
| *VSTM2B* |
| *VWC2* |
| *WBSCR17* |
| *WDR66* |
| *WT1* |
| *WT1-AS* |
| *XDH* |
| *XKR4* |
| *ZBTB8OSP2* |
| *ZFP30* |
| *ZFP42* |
| *ZG16* |
| *ZIC1* |
| *ZIC5* |
| *ZNF135* |
| *ZNF154* |
| *ZNF208* |
| *ZNF32-AS3* |
| *ZNF382* |
| *ZNF454* |
| *ZNF484* |
| *ZNF492* |
| *ZNF529* |
| *ZNF540* |
| *ZNF542* |
| *ZNF565* |
| *ZNF573* |
| *ZNF578* |
| *ZNF667* |
| *ZNF728* |
| *ZNF729* |
| *ZNF732* |
| *ZNF781* |
| *ZNF835* |
| *ZNF876P* |
| *ZNF98* |
| *ZSCAN1* |
| *ZSCAN18* |
| *ZSCAN5A* |

**Supplementary Table 5.** Hypermethylated and down-regulated genes.

| **Gene** | **GO annotations** |
| --- | --- |
| *ACY3* | viral reproduction; xenobiotic metabolic process |
| *ADH1A* | alcohol metabolic process; drug metabolic process; ethanol oxidation; retinoic acid metabolic process; retinol metabolic process |
| *ALDOB* | cellular response to extracellular stimulus; cellular response to insulin stimulus; fructose 1,6-bisphosphate metabolic process; fructose metabolic process; gluconeogenesis; glycolysis; liver development; NADH oxidation; positive regulation of ATPase activity; response to amino acid stimulus; response to cAMP; response to carbohydrate stimulus; response to copper ion; response to drug; response to fructose stimulus; response to glucocorticoid stimulus; response to interleukin-6; response to organic cyclic compound; response to peptide hormone stimulus; response to starvation; response to zinc ion; vacuolar proton-transporting V-type ATPase complex assembly |
| *AMY2A* | carbohydrate catabolic process; carbohydrate metabolic process; metabolic process; polysaccharide digestion; response to bacterium |
| *APOBEC2* | cytidine deamination; cytidine to uridine editing; DNA demethylation; mRNA modification; mRNA processing |
| *AQP12A* | transmembrane transport |
| *AQP8* | canalicular bile acid transport; cellular response to cAMP; transmembrane transport; water transport |
| *ARHGDIG* | blastocyst hatching; negative regulation of cell adhesion; positive regulation of GTPase activity; regulation of protein localization; regulation of small GTPase mediated signal transduction; Rho protein signal transduction |
| *BCAT1* | branched chain family amino acid biosynthetic process; branched chain family amino acid catabolic process; branched chain family amino acid metabolic process; cellular amino acid biosynthetic process; G1/S transition of mitotic cell cycle; leucine biosynthetic process; valine biosynthetic process |
| *C10ORF53* | Unknown |
| *C5* | activation of MAPK activity; cell chemotaxis; cell surface receptor signaling pathway; chemotaxis; complement activation, alternative pathway; complement activation, classical pathway; cytolysis; G-protein coupled receptor signaling pathway; immune system process; inflammatory response; innate immune response; in utero embryonic development; negative regulation of endopeptidase activity; negative regulation of macrophage chemotaxis; positive regulation of angiogenesis; positive regulation of chemokine secretion; positive regulation vascular endothelial growth factor production; regulation of complement activation |
| *CDK5RAP3* | apoptotic nuclear change; brain development; cell proliferation; endoplasmic reticulum unfolded protein response; mitotic cell cycle G2/M transition DNA damage checkpoint; negative regulation of MAP kinase activity; negative regulation of NF-kappaB transcription factor activity; negative regulation of protein kinase activity by regulation of protein phosphorylation; negative regulation of protein phosphorylation; negative regulation of protein serine/threonine kinase activity; positive regulation of protein localization to nucleus; positive regulation of protein ubiquitination; positive regulation of protein ubiquitination involved in ubiquitin-dependent protein catabolic process; positive regulation of transcription from RNA polymerase II promoter; protein ufmylation; regulation of cyclin-dependent protein kinase activity; regulation of mitotic cell cycle; regulation of neuron differentiation; regulation of phosphatase activity |
| *CEL* | ceramide catabolic process; cholesterol catabolic process; fatty acid catabolic process; intestinal cholesterol absorption; intestinal lipid catabolic process; lipid catabolic process; lipid digestion; lipid metabolic process; neuron cell-cell adhesion; pancreatic juice secretion; postsynaptic membrane assembly; presynaptic membrane assembly; protein esterification; regulation of synaptic transmission; retinol metabolic process; synaptic vesicle endocytosis |
| *CLPS* | digestion; lipid catabolic process; lipid digestion; lipid metabolic process; positive regulation of catalytic activity; post-embryonic development; response to bacterium; response to food; retinoid metabolic process |
| *CNTN6* | axon guidance; cell adhesion; central nervous system development; dendrite self-avoidance; homophilic cell adhesion; nervous system development; neurogenesis; neuron differentiation; Notch signaling pathway; positive regulation of Notch signaling pathway |
| *CPA1* | proteolysis; proteolysis involved in cellular protein catabolic process |
| *CPA2* | proteolysis; vacuolar protein catabolic process |
| *CPA4* | histone acetylation; proteolysis |
| *CTRB1* | cobalamin metabolic process; digestion; extracellular matrix disassembly; proteolysis |
| *CTRB2* | cobalamin metabolic process; digestion; extracellular matrix disassembly; positive regulation of apoptotic process; protein catabolic process; proteolysis; response to cytokine stimulus; response to food; response to nutrient; response to peptide hormone stimulus; response to toxin |
| *CTRC* | cellular calcium ion homeostasis; cobalamin metabolic process; proteolysis |
| *CTRL* | protein catabolic process; proteolysis |
| *DCK* | deoxycytidine metabolic process; deoxyribonucleoside monophosphate biosynthetic process; drug metabolic process; nucleobase-containing compound metabolic process; nucleotide biosynthetic process; phosphorylation; purine-containing compound salvage; pyrimidine nucleoside salvage; pyrimidine nucleotide metabolic process |
| *EEF1D* | cellular response to ionizing radiation; mRNA transcription; positive regulation of I-kappaB kinase/NF-kappaB cascade; regulation of cell death; translation; translational elongation |
| *F11* | blood coagulation; blood coagulation, intrinsic pathway; hemostasis; plasminogen activation; positive regulation of fibrinolysis; proteolysis; regulation of blood coagulation |
| *FAM107A* | actin filament bundle assembly; actin filament polymerization; cell cycle; cellular response to glucocorticoid stimulus; cellular response to nutrient levels; cognition; negative regulation of focal adhesion assembly; negative regulation of G1/S transition of mitotic cell cycle; negative regulation of long-term synaptic potentiation; positive regulation of cell migration; positive regulation of protein ubiquitination; regulation of actin cytoskeleton organization; regulation of cell growth; regulation of growth; regulation of microtubule cytoskeleton organization; regulation of protein stability |
| *FBXW12* | post-translational protein modification; protein polyubiquitination |
| *GP2* | antigen transcytosis by M cells in mucosal-associated lymphoid tissue |
| *GPAM* | acyl-CoA metabolic process; CDP-diacylglycerol biosynthetic process; cellular lipid metabolic process; cellular response to insulin stimulus; defense response to virus; fatty acid homeostasis; fatty acid metabolic process; glycerol-3-phosphate metabolic process; glycerophospholipid metabolic process; interleukin-2 secretion; lipid metabolic process; negative regulation of activation-induced cell death of T cells; phosphatidic acid biosynthetic process; phospholipid biosynthetic process; phospholipid homeostasis; positive regulation of activated T cell proliferation; positive regulation of multicellular organism growth; positive regulation of triglyceride biosynthetic process; regulation of cholesterol biosynthetic process; regulation of cytokine secretion; regulation of intracellular estrogen receptor signaling pathway; response to activity; response to cadmium ion; response to fructose stimulus; response to glucose stimulus; response to nutrient levels; triglyceride biosynthetic process; triglyceride metabolic process |
| *GPHA2* | adenylate cyclase-activating G-protein coupled receptor signaling pathway; cell surface receptor signaling pathway; G-protein coupled receptor signaling pathway; regulation of receptor activity |
| *GPT* | Unknown |
| *GSTA5* | glutathione metabolic process; response to bacterium; response to stilbenoid; xenobiotic catabolic process; xenobiotic metabolic process |
| *GUCA1C* | positive regulation of guanylate cyclase activity; regulation of rhodopsin mediated signaling pathway; signal transduction; visual perception |
| *IL22RA1* | cytokine-mediated signaling pathway; defense response to Gram-negative bacterium |
| *KCNJ16* | ion transport; potassium ion transmembrane transport; potassium ion transport; regulation of ion transmembrane transport |
| *KIRREL2* | cell adhesion; negative regulation of protein phosphorylation |
| *KLB* | carbohydrate metabolic process; fibroblast growth factor receptor signaling pathway; MAPK cascade; positive regulation of cell proliferation; positive regulation of MAPKKK cascade by fibroblast growth factor receptor signaling pathway; positive regulation of protein kinase B signaling cascade |
| *LMO3* | negative regulation of ERK1 and ERK2 cascade; positive regulation of fat cell differentiation; positive regulation of glucocorticoid receptor signaling pathway; positive regulation of peroxisome proliferator activated receptor signaling pathway |
| *MARVELD2* | cell-cell junction organization; establishment of endothelial barrier; sensory perception of sound; tight junction assembly |
| *MAT1A* | methionine catabolic process; methylation; one-carbon metabolic process; protein homooligomerization; protein homotetramerization; protein tetramerization; S-adenosylmethionine biosynthetic process; selenium compound metabolic process; sulfur amino acid metabolic process |
| *MEP1A* | proteolysis |
| *MYLK2* | cardiac muscle cell differentiation; cardiac muscle contraction; cardiac muscle tissue morphogenesis; muscle contraction; neuromuscular synaptic transmission; peptidyl-serine phosphorylation; peptidyl-threonine phosphorylation; phosphorylation; positive regulation of fast-twitch skeletal muscle fiber contraction; positive regulation of gene expression; protein autophosphorylation; protein phosphorylation; regulation of cell cycle; regulation of MAPK cascade; regulation of muscle contraction; regulation of muscle filament sliding; regulation of neuronal synaptic plasticity; satellite cell differentiation; skeletal muscle cell differentiation; striated muscle contraction; synaptic vesicle transport; water transport |
| *MYOM1* | ardiac muscle fiber development; cardiac muscle tissue morphogenesis; cardiac myofibril assembly; extraocular skeletal muscle development; muscle contraction; positive regulation of gene expression; positive regulation of protein secretion; protein kinase A signaling cascade; sarcomere organization; skeletal muscle myosin thick filament assembly; skeletal muscle thin filament assembly; striated muscle myosin thick filament assembly |
| *NPTX2* | associative learning; neuron projection development; synaptic transmission |
| *NR0B2* | cholesterol metabolic process; circadian regulation of gene expression; circadian rhythm; negative regulation of gene expression; negative regulation of sequence-specific DNA binding transcription factor activity; negative regulation of transcription, DNA-dependent; negative regulation of transcription from RNA polymerase II promoter; Notch signaling pathway; organ regeneration; positive regulation of gene expression; positive regulation of insulin secretion; regulation of transcription, DNA-dependent; response to glucose stimulus; rhythmic process; steroid hormone mediated signaling pathway; transcription initiation from RNA polymerase II promoter |
| *NUPR1* | acute inflammatory response; cell proliferation; DNA damage response, signal transduction by p53 class mediator resulting in induction of apoptosis; macromolecular complex assembly; male gonad development; negative regulation of apoptotic process; negative regulation of autophagy; negative regulation of cardiac muscle cell apoptotic process; negative regulation of cell cycle; negative regulation of cell proliferation; negative regulation of epithelial cell proliferation; negative regulation of fibroblast proliferation; negative regulation of glycolysis; negative regulation of sequence-specific DNA binding transcription factor activity; positive regulation of apoptotic process; positive regulation of catalytic activity; positive regulation of intrinsic apoptotic signaling pathway; positive regulation of neuron apoptotic process; positive regulation of protein modification process; protein acetylation; regulation of autophagy; regulation of female gonad development; response to toxin; skeletal muscle cell differentiation |
| *PAH* | aromatic amino acid family metabolic process; catecholamine biosynthetic process; cellular amino acid biosynthetic process; L-phenylalanine catabolic process; L-phenylalanine metabolic process; metabolic process; neurotransmitter biosynthetic process; oxidation-reduction process; protein hydroxylation; pteridine-containing compound metabolic process; tetrahydrobiopterin metabolic process; tyrosine biosynthetic process |
| *PCDHGB6* | ell adhesion; homophilic cell adhesion |
| *PDIA2* | cell redox homeostasis; oxidation-reduction process; protein folding; protein folding in endoplasmic reticulum; protein retention in ER lumen; response to endoplasmic reticulum stress |
| *PDP2* | carbohydrate utilization; peptidyl-threonine dephosphorylation; protein dephosphorylation; regulation of acetyl-CoA biosynthetic process from pyruvate |
| *PHYHD1* | oxidation-reduction process |
| *PLA2G1B* | actin filament organization; activation of MAPK activity; activation of phospholipase A2 activity; antibacterial humoral response; arachidonic acid secretion; cellular response to insulin stimulus; defense response to Gram-positive bacterium; fatty acid biosynthetic process; innate immune response in mucosa; interleukin-1 production; interleukin-8 production; intracellular signal transduction; leukotriene biosynthetic process; lipid catabolic process; lipid metabolic process; neutrophil chemotaxis; neutrophil mediated immunity; phosphatidic acid biosynthetic process; phosphatidylcholine acyl-chain remodeling; phosphatidylcholine metabolic process; phosphatidylethanolamine acyl-chain remodeling; phosphatidylglycerol acyl-chain remodeling; phosphatidylinositol acyl-chain remodeling; phosphatidylserine acyl-chain remodeling; phospholipid catabolic process; phospholipid metabolic process; positive regulation of calcium ion transport into cytosol; positive regulation of cell proliferation; positive regulation of fibroblast proliferation; positive regulation of immune response; positive regulation of NF-kappaB transcription factor activity; positive regulation of protein secretion; positive regulation of transcription from RNA polymerase II promoter; regulation of glucose import; signal transduction |
| *PNLIP* | intestinal cholesterol absorption; lipid catabolic process; lipid digestion; lipid metabolic process; positive regulation of triglyceride lipase activity; post-embryonic development; response to lipid; response to peptide hormone stimulus; retinoid metabolic process |
| *PNLIPRP1* | lipid catabolic process; lipid metabolic process; pancreas development; response to glucocorticoid stimulus; response to peptide hormone stimulus |
| *PNLIPRP2* | cellular defense response; galactolipid catabolic process; intestinal lipid catabolic process; lipid catabolic process; lipid digestion; lipid metabolic process; phospholipid catabolic process; post-embryonic development; response to bacterium; response to food; response to glucocorticoid stimulus; response to lipid; response to peptide hormone stimulus; triglyceride metabolic process |
| *PRSS1* | cobalamin metabolic process; digestion; extracellular matrix disassembly; proteolysis |
| *PRSS8* | cornification; hair follicle development; positive regulation of sodium ion transport; proteolysis; response to drug; response to mineralocorticoid stimulus; response to peptide hormone stimulus; transepithelial transport |
| *RAB26* | exocrine system development; Golgi to plasma membrane protein transport; intracellular protein transport; protein transport; Rab protein signal transduction; regulated secretory pathway; regulation of exocytosis; vesicle-mediated transport |
| *REG1A* | calcium ion homeostasis; cell wall disassembly in other organism; midgut development; negative regulation of cell proliferation; positive regulation of cell proliferation; positive regulation of gene expression; protein homooligomerization; protein homotetramerization; regulation of receptor activity; response to hypoxia; response to nutrient levels; response to organic cyclic compound; response to peptide hormone stimulus; signal transduction; wound healing |
| *RNF186* | intrinsic apoptotic signaling pathway in response to endoplasmic reticulum stress; proteasomal ubiquitin-dependent protein catabolic process; protein autoubiquitination; protein K29-linked ubiquitination; protein K63-linked ubiquitination; protein localization in mitochondrion |
| *RORC* | adipose tissue development; alpha-beta T cell differentiation; cell differentiation; circadian regulation of gene expression; cytokine-mediated signaling pathway; interleukin-17 production; interleukin-17 secretion; intracellular receptor mediated signaling pathway; lymph node development; mucosal-associated lymphoid tissue development; multicellular organismal development; negative regulation of thymocyte apoptotic process; negative regulation of transcription from RNA polymerase II promoter; Peyer's patch development; positive regulation of circadian rhythm; positive regulation of transcription, DNA-dependent; protein phosphorylation; regulation of fat cell differentiation; regulation of gamma-delta T cell differentiation; regulation of glucose metabolic process; regulation of steroid metabolic process; regulation of transcription, DNA-dependent; regulation of transcription involved in cell fate commitment; rhythmic process; steroid hormone mediated signaling pathway; T cell differentiation in thymus; T-helper 17 cell differentiation; T-helper cell differentiation; transcription initiation from RNA polymerase II promoter; xenobiotic metabolic process |
| *RYR2* | BMP signaling pathway; calcium ion transmembrane transport; calcium ion transport; calcium ion transport into cytosol; calcium-mediated signaling; calcium-mediated signaling using intracellular calcium source; canonical Wnt receptor signaling pathway; cardiac muscle contraction; cardiac muscle hypertrophy; cell communication by electrical coupling involved in cardiac conduction; cellular calcium ion homeostasis; cellular response to caffeine; cellular response to epinephrine stimulus; cytosolic calcium ion homeostasis; cytosolic calcium ion transport; detection of calcium ion; embryonic heart tube morphogenesis; establishment of protein localization in endoplasmic reticulum; ion transmembrane transport; ion transport; left ventricular cardiac muscle tissue morphogenesis; manganese ion transmembrane transport; multicellular organismal development; positive regulation of heart rate; positive regulation of sequestering of calcium ion; Purkinje myocyte to ventricular cardiac muscle cell signaling; reduction of cytosolic calcium ion concentration; regulation of cardiac muscle contraction; regulation of cardiac muscle contraction by calcium ion signaling; regulation of cardiac muscle contraction by regulation of the release of sequestered calcium ion; regulation of heart rate; regulation of ventricular cardiac muscle cell action potential; release of sequestered calcium ion into cytosol; release of sequestered calcium ion into cytosol by sarcoplasmic reticulum; response to caffeine; response to calcium ion; response to drug; response to hypoxia; response to magnesium ion; response to muscle activity; response to muscle stretch; response to nutrient; response to redox state; sarcoplasmic reticulum calcium ion transport; transmembrane transport; type B pancreatic cell apoptotic process |
| *SALL3* | forelimb morphogenesis; hindlimb morphogenesis; negative regulation of smoothened signaling pathway; olfactory bulb interneuron development; regulation of transcription, DNA-dependent; regulation of transcription from RNA polymerase II promoter |
| *SLC9A4* | cation transport; epithelial cell development; gastric acid secretion; ion transport; potassium ion transmembrane transport; regulation of intracellular pH; regulation of pH; sodium ion transmembrane transport; sodium ion transport; transmembrane transport |
| ***SST*** | **cell-cell signaling; cell surface receptor signaling pathway; digestion; G-protein coupled receptor signaling pathway; hyperosmotic response; induction of apoptosis by hormones; negative regulation of cell proliferation; regulation of cell migration; regulation of receptor activity; response to acidity; response to amino acid stimulus; response to drug; response to heat; response to nutrient; response to organic nitrogen; response to steroid hormone stimulus; synaptic transmission** |
| *STX19* | exocytosis; intracellular protein transport; synaptic vesicle fusion to presynaptic membrane; vesicle docking; vesicle fusion; vesicle-mediated transport |
| *TMEM125* | unknown |
| *UGT2A3* | cellular glucuronidation |
| *VIPR2* | activation of adenylate cyclase activity; cell-cell signaling; cell surface receptor signaling pathway; G-protein coupled receptor signaling pathway; negative regulation of smooth muscle cell proliferation; signal transduction |
| *ZG16* | protein transport |
